# The FOXP2-Driven Network in Developmental Disorders and Neurodegeneration

**DOI:** 10.3389/fncel.2017.00212

**Published:** 2017-07-26

**Authors:** Franz Oswald, Patricia Klöble, André Ruland, David Rosenkranz, Bastian Hinz, Falk Butter, Sanja Ramljak, Ulrich Zechner, Holger Herlyn

**Affiliations:** ^1^Center for Internal Medicine, Department of Internal Medicine I, University Medical Center Ulm Ulm, Germany; ^2^Institut für Organismische und Molekulare Evolutionsbiologie, Johannes Gutenberg-University Mainz Mainz, Germany; ^3^Institute of Human Genetics, University Medical Center Mainz Mainz, Germany; ^4^Institute of Molecular Biology Mainz, Germany; ^5^Sciema UG Mainz, Germany; ^6^Dr. Senckenbergisches Zentrum für Humangenetik Frankfurt, Germany

**Keywords:** language, speech, brain, schizophrenia, Parkinson’s disease, Alzheimer’s disease, Huntington’s disease, neuronal circuitry

## Abstract

The transcription repressor FOXP2 is a crucial player in nervous system evolution and development of humans and songbirds. In order to provide an additional insight into its functional role we compared target gene expression levels between human neuroblastoma cells (SH-SY5Y) stably overexpressing *FOXP2* cDNA of either humans or the common chimpanzee, Rhesus monkey, and marmoset, respectively. RNA-seq led to identification of 27 genes with differential regulation under the control of human *FOXP2*, which were previously reported to have FOXP2-driven and/or songbird song-related expression regulation. RT-qPCR and Western blotting indicated differential regulation of additional 13 new target genes in response to overexpression of human *FOXP2.* These genes may be directly regulated by FOXP2 considering numerous matches of established FOXP2-binding motifs as well as publicly available FOXP2-ChIP-seq reads within their putative promoters. Ontology analysis of the new and reproduced targets, along with their interactors in a network, revealed an enrichment of terms relating to cellular signaling and communication, metabolism and catabolism, cellular migration and differentiation, and expression regulation. Notably, terms including the words “neuron” or “axonogenesis” were also enriched. Complementary literature screening uncovered many connections to human developmental (autism spectrum disease, schizophrenia, Down syndrome, agenesis of corpus callosum, trismus-pseudocamptodactyly, ankyloglossia, facial dysmorphology) and neurodegenerative diseases and disorders (Alzheimer’s, Parkinson’s, and Huntington’s diseases, Lewy body dementia, amyotrophic lateral sclerosis). Links to deafness and dyslexia were detected, too. Such relations existed for single proteins (e.g., DCDC2, NURR1, PHOX2B, MYH8, and MYH13) and groups of proteins which conjointly function in mRNA processing, ribosomal recruitment, cell–cell adhesion (e.g., CDH4), cytoskeleton organization, neuro-inflammation, and processing of amyloid precursor protein. Conspicuously, many links pointed to an involvement of the FOXP2-driven network in JAK/STAT signaling and the regulation of the ezrin–radixin–moesin complex. Altogether, the applied phylogenetic perspective substantiated FOXP2’s importance for nervous system development, maintenance, and functioning. However, the study also disclosed new regulatory pathways that might prove to be useful for understanding the molecular background of the aforementioned developmental disorders and neurodegenerative diseases.

## Introduction

**FIGURE 1:**
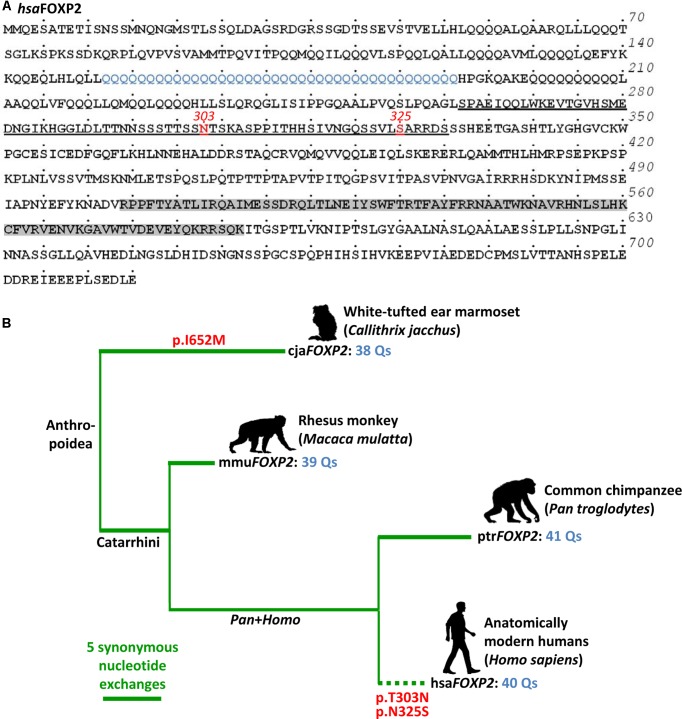
Evolution of *FOXP2* in primates (Anthropoidea). **(A)** Human FOXP2 protein (*hsa*FOXP2: NP_055306) with two characteristic amino acids at positions 303 and 325 highlighted in red. Underlining broadly defines the transcription repression domain. The FOX domain is shown in gray (domains after Zhang et al., 2002). Blue labeling highlights the longest poly-glutamine stretch within the glutamine-enriched N-terminus. Dots above the amino acid sequence indicate increments of five amino acids, each. Numbers on the right give the total number of amino acids. **(B)** *FOXP2* gene tree (Primates, Anthropoidea). Horizontal branch lengths (thick green lines) correspond to the number of synonymous exchanges. Red labels indicate two non-synonymous exchanges, which occurred on the human branch (dotted line). The respective amino acid exchanges occured inside the transcription repression domain, as shown in **(A)**. Another non-synonymous exchange took place on the lineage to the marmoset. A detailed list of the branch-specific exchanges is given in Supplementary Table 1.1. *FOXP2* cDNAs (cja*FOXP2* etc.) are designated according to the Latin species names (cja, *Callithrix jacchus* etc.).

The high complexity clearly sets apart human verbal communication from vocalization repertoires of other primate species. Yet, despite this importance we are still at the beginning of understanding the molecular pathways behind the evolutionary and developmental acquisition of speech and language. Probably, the most advancement was made in respect to the role of the gene coding for forkhead box P2 (FOXP2; O15409; also CAGH44). The encoded transcription repressor spans 715 amino acids (aa) in human isoform I, thereby containing the eponymous forkhead box domain with DNA-binding ability at the C-terminus, a central expression suppression domain with zinc finger and leucine zipper motifs, and a glutamine-enriched N-terminus, with the longest poly-glutamine stretch spanning 40 aa (**Figure 1A**; e.g., Vernes and Fisher, 2009; Enard, 2011).

FOXP2’s relevance for verbal communication became obvious when a missense mutation in the coding gene (p.R553H) which lowers the DNA binding capability was recognized to associate with speech-language disorder 1 (SPCH1, OMIM #602081), also known as developmental verbal dyspraxia (DVD) and childhood apraxia of speech (CAS). Affected family members suffered from deficits in virtually every aspect of expressive and receptive language. They especially showed disturbed orofacial motor coordination affecting tongue, lips, jaw and palate, which together led to impaired lingual articulation and non-lingual sound-production (e.g., Vernes and Fisher, 2009). The subsequent recognition of associations between other *FOXP2* mutations and communication disorders further substantiated the importance of the gene for the acquisition of full speech and language competence (Vernes and Fisher, 2009; Palka et al., 2012). *FOXP2* has additionally been implicated in the etiology of mental diseases such as autism spectrum disorder (ASD; Bowers and Konopka, 2012) and schizophrenia (SCZD; e.g., Jamadar et al., 2011; Li et al., 2013). Notably, also these disorders are frequently accompanied by language and speech deficits (e.g., Stephane et al., 2007; Abrahams and Geschwind, 2010; Kupferberg, 2010) so that studies on *FOXP2* hold out the prospect of elucidating the evolution and development of speech and language (e.g., Marcus and Fisher, 2003; Bolhuis et al., 2010; Enard, 2011).

The *FOXP2* gene is expressed in multiple tissues including fetal and adult brain (e.g., Vernes and Fisher, 2009; Enard, 2011) whereby haploinsufficiency and thus lowered levels of transcript and protein are commonly assumed to elicit the aforementioned diseases and disorders (e.g., Enard, 2011). In support of this view, all documented patients were heterozygous for the etiological mutation (Vernes and Fisher, 2009) in either the gene itself (point mutations, deletions, chromosomal rearrangements; e.g., Turner et al., 2013 and references therein) or downstream regulatory elements (e.g., Adegbola et al., 2015). Random mono-allelic expression (RMAE) with some cells having half the *FOXP2* dosage and others expressing none at all (Adegbola et al., 2015) and mosaic deletion with some cells possessing two functional alleles and others none (Palka et al., 2012) also play a role. Either way, minimum expression of one functional allele in at least part of the cells seems indispensable to life, a condition that is additionally demonstrated by early post-natal death of mice homozygous for a *Foxp2* null allele (French et al., 2007; Vernes et al., 2007; Rousso et al., 2012).

FOXP2’s influence on human vocalization skills has a stunning parallel in non-primate vocal-learners. In songbirds, brain *FOXP2* levels positively associate with vocal learning and singing activity whereas knockdown impairs song learning (see, e.g., Bolhuis et al., 2010; Pfenning et al., 2014). In line with this, *FOXP2* belonged to the highly supported genes in a genome-wide screen for singing-related transcriptional changes in male zebra finch brains (see Hilliard et al., 2012). Investigation of other bird and additional mammalian species underlined a general pattern confirming that normal development of vocal learning and vocalization skills requires fine-tuned regulation of FOXP2 expression in brain (e.g., Bolhuis et al., 2010; Pfenning et al., 2014).

The human–bird parallel demonstrates that FOXP2’s origin predates the split of Mammalia and Sauropsida about 320 million years ago (timetree.org estimate). Yet, regardless of hundreds of millions of years of independent evolution zebra finch FOXP2 still shows 98% identity with the human ortholog (Haesler et al., 2004). Evolutionary conservation of FOXP2 also prevailed throughout the divergence of primates, though with a notable exception: Thus, two aa substitutions, p.T303N and p.N325S (rs753394697 SNP), occurred on the human branch after the split from the chimpanzee lineage. These two exchanges reside inside the transcription repression domain (**Figure 1A**) and potentially were exposed to positive selection (reviewed in Enard, 2011; also, e.g., Mozzi et al., 2016). However, according to genome-wide evidence the selective sweep was not complete (Mallick et al., 2016) so that *FOXP2* evolution in primates and especially in humans was probably more complex than previously thought. In any case, the two aa exchanges known from humans seem to be functional. Thus, mice homozygous for a *Foxp2* version mutated for both human exchanges had reduced dopamine concentration in all investigated brain regions and their striatal medium spiny neurons showed increased dendrite length, synaptic plasticity, and long term depression (Enard et al., 2009). A related study substantiated the relevance of both aa exchanges for dendrite length of neurons in cortico-basal ganglia circuits (Reimers-Kipping et al., 2011).

The investigation of how mutations that associate with verbal dyspraxia affect FOXP2 expression, subcellular localization, DNA-binding and transactivation properties in human neuron-related cells (SH-SY5Y) inspired a series of further *in vitro* analyses (Vernes et al., 2006). Two of the sequel studies identified diverse FOXP2 target candidates in SH-SY5Y cells that were transfected with either human *FOXP2* cDNA or empty vector (Spiteri et al., 2007; Vernes et al., 2007). Based on the same *in vitro* approach, one of the follow-up studies determined retinoic acid signaling as an important pathway in FOXP2-driven neuronal differentiation (Devanna et al., 2014). Another investigation addressed potential regulatory changes in humans by comparing target gene expression levels between SH-SY5Y cells overexpressing either human *FOXP2* cDNA or a variant in which both human-specific non-synonymous substitutions were mutated to acquire the chimpanzee-specific aa content at the respective sites (*FOXP2^chimp^* in Konopka et al., 2009). The latter study as well as additional ones with a focus on the effect of both human-specific aa substitutions in mutated mice (*Foxp2^hum^* in Enard et al., 2009 and follow-up studies) generated valuable data on FOXP2/Foxp2 functioning. However, the interpretability of the data is fairly challenging from an evolutionary point of view, due to the following: Understanding the evolutionary meaning behind differential target gene expression levels in a pair of models representing two extant species requires considerable assumptions in regard to expression levels in an unknown ancestor. Without such assumptions it is not possible to certainly assign an evolutionary change to either one or the other lineage. Neither parental cells nor cells carrying empty vector can appropriately model the ancestral condition as they rather reflect baseline expression levels in extant species as do wild-type animals in respective comparisons. Moreover, the mutated *FOXP2*/*Foxp2* variants which merge states of two species in a single cDNA (*FOXP2^chimp^*, *Foxp2^hum^*) have no counterparts in living species and it is questionable if they have ever existed in any human ancestor. This is due to additional synonymous (silent) and non-synonymous nucleotide substitutions in the human–mouse and human–chimpanzee comparison (see, e.g., Enard et al., 2002). Variant lengths of the CAG/CAA repeats coding for the N-terminal FOXP2 poly-glutamine tracts additionally contribute to this discrepancy (**Figure 1A**). Yet, the outlined restraints in terms of evolutionary interpretability can be overcome in a broader phylogenetic context which addresses the effect of human *FOXP2* relative to naturally occurring cDNAs of chimpanzee and at least one further non-human species. In such a phylogenetic approach unidirectional differences in target gene expression levels between the human model on the one hand and the non-human models on the other give an approximation of potential expressional changes in human evolution.

Synonymous exchanges might indeed have functional relevance on FOXP2 expression, namely through nucleotide (Debatisse et al., 2004) and tRNA availability (Wohlgemuth et al., 2013). It is also conceivable that extension of the longest of N-terminal poly-glutamine tracts is functionally relevant. The respective stretch spans 40 aa in the human reference (*Homo sapiens* FOXP2, hsaFOXP2) whereas it has 41, 39, and 38 glutamines in the FOXP2 of common chimpanzee (*Pan troglodytes*, ptrFOXP2), Rhesus monkey (*Macaca mulatta*, mmuFOXP2), and white-tufted ear marmoset (*Callithrix jacchus*, cjaFOXP2), respectively (**Figure 1B**). Although these interspecific differences may appear negligible they seem to evolve under functional constraint as suggested by a general tendency of repeat length conservation in humans (Bruce and Margolis, 2002). In support of this view, mutations decreasing the number of N-terminal glutamines in FOXP2 were found to occur in speech and sound disorder (SSD) patients. Also, the fact that a deletion of glutamines from the FOXP2 N-terminus alters expression of the language gene *CNTNAP2* suggests that the extension of the respective stretches is functionally relevant (Zhao et al., 2015; for language association, see Abrahams and Geschwind, 2010; Kato et al., 2014). Functional relevance of poly-glutamine tract extension could be anticipated given that poly-glutamine tract expansion in other genes account for several diseases (e.g., Fan et al., 2014). On that premise, some of FOXP2’s functional implications in human evolution and development might still await their discovery.

The present study investigates FOXP2’s role by adopting a broader phylogenetic perspective. To the best of our knowledge, this approach takes into account for the first time the entire spectrum of differences that distinguish human *FOXP2* gene from its non-human primate counterparts. In detail, we compared expression levels between SH-SY5Y cells stably overexpressing hsa*FOXP2* with corresponding levels in cells that were alternatively transfected with ptr*FOXP2*, mmu*FOXP2*, and cja*FOXP2*. The species sample behind covers the major lineages inside extant anthropoid primates (New World monkeys, Old World monkeys, and Hominoidea) and at the same time allows for the identification of changes on the human branch (for phylogenetic relationships, see **Figure 1B**). We investigated which of the genes with specific expression regulation under hsa*FOXP2* control already showed FOXP2/Foxp2-driven and/or songbird song-related expression regulation in previous studies. Additional attention was payed to the question if the FOXP2-driven network might be more comprehensive than known so far. We finally addressed which of the functional implications of the proteins in our network confirm previous knowledge, and which pathways might have been not observed before.

## Materials and Methods

### Cell Culture and Transfection

We evaluated pcDNA3-constructs in HEK293 human embryonic kidney cells (ATCC no. CRL-1573) that were cultivated in DMEM (Gibco) supplemented with 10% FCS (Biochrom) and 1% Penicillin–Streptomycin (Gibco) at 37°C and 5% CO_2_. After RT-PCR detected only minimum amount of endogenous *FOXP2* transcript (Supplementary Image 1), cells (1 × 10^6^ cells, seeded in 8 cm dishes) were transiently transfected with Nanofectin (PAA) with 8 μg of either empty pcDNA3 expression vector (Thermo Fisher) or constructs carrying alternative primate *FOXP2* cDNAs. The custom-synthesized (BlueHeron) cDNAs used for transfection were species-specific and coded for human FOXP2 isoform I (715 aa; ENST00000350908; *Homo sapiens*, hsa) and FOXP2s in common chimpanzee (AY064549; *Pan troglodytes*, ptr), Rhesus monkey (ENSMMUT00000011202; *Macaca mulatta*, mmu), and white-tufted ear marmoset (XM_002751707; *Callithrix jacchus*, cja). Full-length transcription and translation of hsa*FOXP2*, ptr*FOXP2*, mmu*FOXP2*, and cja*FOXP2* was verified through specific molecular weights (plus/minus FLAG tag: higher/lower molecular weight) in Western blots 24 h after transfection (Supplementary Image 2). For the subsequent generation of stable transfectants we used *FOXP2*-specific expression plasmids without N-terminal FLAG tag.

Parental SH-SY5Y neuroblastoma cells (ATCC no. CRL-2266) were grown in DMEM containing 15% FCS and 1% Penicillin–Streptomycin. After RT-PCR (Supplementary Image 1) detected no endogenous *FOXP2* transcript, SH-SY5Y cells were transfected with 2 μg of either linearized empty plasmid (pcDNA3) or *FOXP2*-specific pcDNA3-constructs (see above), using the Amaxa Cell Line Nucleofector V Kit (Lonza) according to the manufacturer’s instructions. This was done three times, thus generating three biological replicates per condition (designated I-III). Cells were cultivated in selection medium supplemented with 600 μg/ml geneticin (G-418; PAA; optimized concentration according to toxicity testing) to enforce stable transfection. Stable expression of FOXP2 protein was repeatedly monitored by Western blotting (see: Immunoblotting). Efficiency and persistence of transfection were additionally monitored in SH-SY5Y cells carrying pcDNA3-eGFP constructs, by fluorescence microscopy and through Western blotting using anti-eGFP (mouse monoclonal IgG, cat 11814460001, Roche; peroxidase conjugated sheep anti-mouse IgG, NA931V, GE Healthcare).

### RT-PCR and Sanger Sequencing

Coding DNAs were generated with SuperScript II (Invitrogen; random primers) from total RNAs extracted with RNeasy Mini Kit (Qiagen). Subsequent standard PCR (Taq DNA Polymerase; Invitrogen) used primers hybridizing to evolutionary conserved sites of *FOXP2* cDNA (forward: 5′-AACAGAGACCACTGCAGGTGCC-3′; reverse: 5′-TCCCTGACGCTGAAGGCTGAG-3′). For assessing levels of endogenous *FOXP2* transcription in parental HEK293 and SH-SY5Y cell lines, PCR reactions were separated on an ethidium bromide-stained agarose gel, documented under UV light, and evaluated by eye. For validating transfection of SH-SY5Y cells with the intended pcDNA3 construct, RT-PCR set the start for subsequent gel extraction of *FOXP2* bands (Gel Extraction Kit, Qiagen), ligation into TOPO vector (TOPO TA, Invitrogen), cloning into *Escherichia coli* XL1-Blue (Stratagene), plasmid preparation (Wizard Plus, Promega), and Sanger sequencing with vector primer M13 (Sequiserve).

### RNA Sequencing

Barcoded mRNA-seq cDNA libraries were prepared from 600 ng of total RNA of biological replicates I and II per each condition, using Illumina’s TruSeq RNA Sample Preparation Kit. mRNA was isolated using oligo(d)T magnetic beads. Isolated mRNA was fragmented using divalent cations and heat and converted into cDNA using random primers and SuperScript II, followed by second strand synthesis. cDNA was end repaired, 3′ adenylated and single T-overhang Illumina multiplex specific adapters were ligated to the cDNA fragments, followed by an enrichment PCR. All cleanups were done using Agencourt AMPure XP magnetic beads. The quantity of the resulting cDNA mRNA-Seq libraries was measured using Qubit. Barcoded mRNA-Seq libraries were clustered on the cBot using the TruSeq PE cluster kit V3 (10 pM) and 2 × 50 bp were sequenced on the Illumina HiSeq 2500 (TruSeq SBS V3 kit; 50 cycles). Raw and processed data of RNA-seq have been deposited at NCBI’s Gene Expression Omnibus (GEO) under accession number GSE100291.

The raw output data of the HiSeq was preprocessed according to the Illumina standard protocol. This includes filtering for low quality reads and demultiplexing. Sequence reads were aligned to the reference genomic sequence (hg19) using STAR^1^. The alignment coordinates were compared to the exon coordinates of the UCSC transcripts^2^ and for each transcript the counts of overlapping alignments were recorded. The read counts were normalized to numbers of bases which map per kb of exon model per million mapped bases (BPKM; see Mortazavi et al., 2008) for each transcript. Comparisons between alternatively transfected cells were conducted on the basis of BPKM values as averaged over the transcripts identified.

### Reverse Transcription Quantitative PCR

Coding DNA was synthesized from 2 μg total RNA of biological replicates I and II (per each condition) by reverse transcription using oligo(d)T and random primers with SuperScript III (Invitrogen) according to the manufacturer’s instructions. The cDNA samples were diluted 1:40, and 7.5 μl of the diluted cDNA was used for reverse transcription quantitative PCR (RT-qPCR) of the candidate genes (for primers, see Supplementary Table 1.2) with QuantiTect SYBR Green Master Mix (Qiagen) on a StepOnePlus Real-Time PCR System (Life Technologies). Data was first explored with LinRegPCR^3^ for calculating PCR efficiency. Subsequently, relative expression was calculated using the 2^-2ΔΔCt^ method (Livak and Schmittgen, 2001). Measurements were carried out thrice per biological replicate. For data normalization, we measured mRNA levels of the reference genes *GAPDH* and *RPLP* (for primers, see Supplementary Table 1.2).

### Immunoblotting

We focused on proteins for which commercially available antibodies yielded specific bands of the expected molecular weight in Western blots. These analyses were carried out on the basis of all three biological replicates that we prepared per condition (I-III). Protein isolation, protein quantification, SDS-PAGE, Western blotting (PVDF, Millipore), blocking and incubation with antibodies, and Enhanced Chemiluminescence (ECL, GE Healthcare) followed standard protocols. FOXP2 protein expression in transiently transfected HEK293 cells was monitored by ECL in Western blots (primary antibody: anti FOXP2 polyclonal goat anti-human, ab1307, Abcam; secondary antibody: peroxidase-conjugated rabbit anti-goat IgG, Jackson ImmunoResearch). Protein levels in stably transfected SH-SY5Y cells (all without FLAG tag) were assessed by Western blotting and ECL using the following antibodies: anti-FOXP2 (monoclonal rabbit anti-human IgG, F9050-02C, Biomol, secondary antibody: peroxidase-conjugated donkey anti-rabbit IgG, NA934V, GE Healthcare), anti-BACE2 (mouse monoclonal IgG, sc271286, Santa Cruz Biotechnology, secondary antibody: peroxidase-conjugated sheep anti-mouse IgG, NA931V, GE Healthcare), anti-MSN (monoclonal rabbit IgG, ab52490, Abcam, secondary antibody: NA934V, GE Healthcare), anti-CDH4 (polyclonal rabbit IgG, sc7941, Santa Cruz Biotechnology, secondary antibody: NA934V, GE Healthcare). The anti-human FOXP2 antibody was raised against a peptide sequence which is conserved across the species included. Beta-actin (β-actin) served as a standard for protein loading (anti-β-actin antibody: mouse monoclonal IgG, A1978, Sigma, secondary antibody: NA931V). For densitometric analysis, signal intensity was scanned at least twice (two technical replicates) from Western blots of three biological replicates using the ImageJ software^4^.

### Bioinformatics and Statistics

Gene and protein symbols accord to the recommendations of the Human Gene Nomenclature Committee.

Branch-specific synonymous (silent) and aa altering exchanges in *FOXP2* were inferred by Codeml, as implemented in the PAML package v. 4.7 (Yang, 2007). For meeting the demands of PAML, we compiled a species tree with three equally ranking branches leading to the zebra finch (*Taeniopygia guttata*), the European house mouse (*Mus musculus*), and the four primate species considered (Anthropoidea). The relationships amongst the four anthropoid species reflected the commonly accepted phylogeny (e.g., Perelman et al., 2011). Codeml analysis additionally used an alignment (ClustalX implemented in BioEdit; Hall, 1999) of the corresponding four anthropoid cDNAs (for accession numbers, see above) and of their murine (ENSMUST00000115477.7) and zebra finch (AY549148.1) orthologs.

We screened BPKM values from RNA-seq for genes whose expression levels differed in the same direction (up-/down) between each of the hsa*FOXP2*-overexpressing SH-SY5Y transfectants and every transfectant overexpressing a non-human primate *FOXP2* cDNA or carrying empty vector. This entry criterion was tightened for new FOXP2 targets which additionally had to show at least twofold differential expression levels between the human and every other tested condition (mean *versus* mean). Statistical significance of expression levels in hsa*FOXP2*-overexpressing *versus* non-human primate *FOXP2*-overexpressing cells was then assessed employing the two-tailed *t*-test in SPSS v. 23.0 (IMB). The same test was applied to relative expression levels (RT-qPCR) and densitometric values (Western blotting).

Expression analyses included an evaluation of the magnitude of the effect, which stable overexpression of hsa*FOXP2* had on target gene transcription and translation in SH-SY5Y cells relative to the alternative treatment with non-human primate *FOXP2* cDNAs. In detail, we calculated the correlation coefficient *r*, thereby taking into account inhomogeneous variances between samples and unequal sample sizes (Cohen, 1988). The *r*-values were also used for *post hoc* analyses of the power of *t*-tests, which were carried out with the aid of G^∗^Power 3.1.9.2 (Faul et al., 2009). Following the convention, we regarded *r*-values of at least 0.5 and power estimates of >80% as approximate benchmarks of large effect size and acceptable test power, respectively (Cohen, 1988).

As detailed in the legend of present Supplementary Table 2.1, we matched our RNA-seq data with previously published lists of potential targets of human FOXP2 and murine Foxp2 as identified by Spiteri et al. (2007, their Table 1), Vernes et al. (2007, their Table 1), Enard et al. (2009, their Figures S8A,B, right panel), Konopka et al. (2009, their Supplementary Table 1), and Vernes et al. (2011, their Table S1). We additionally checked our data for matches with genes that showed singing-related expression regulation in zebra finch brain (Hilliard et al., 2012, their Table S2: only genes where *q*-values indicated significant support).

In addition, we mapped publicly available FOXP2-binding sequences on putative promoter sequences (5,000 bp upstream of transcription start) of the newly defined FOXP2 candidate genes. The down-loaded sequences were generated by chromatin immunoprecipitation with an antibody against 127 C-terminal aa of human FOXP2, followed by sequencing (FOXP2-ChIP-seq). The respective DNA was isolated from human neuroblastoma SK-N-MC cells (GEO project GSM803353: SRR351544; see also Nelson et al., 2013). The mapping results were normalized for the number of hits across the human genome (GRCh38.p7). The putative promoter sequences of the same genes were also screened for established FOXP2-binding motifs (see Stroud et al., 2006; Vernes et al., 2007, 2011; Nelson et al., 2013). This was done with the aid of SeqMap v. 1.0.3 (Jiang and Wong, 2008), without allowing for any mismatch.

Following others (e.g., Boeckx and Benítez-Burraco, 2014a,b) we employed the STRING server (v. 10.0^5^) for the reconstruction of a protein–protein interaction (PPI) network as well as for PPI enrichment and gene ontology (GO) enrichment analyses. Thresholds for the acceptance of a PPI were alternatively set to low (≥0.15), medium (≥0.4), high (≥0.7), and maximum combined confidence scores (≥0.9). We inferred node degree values per protein (= number of direct edges a protein has) with the aid of Cytoscape v. 3.2.1 and the plugin NetworkAnalyzer 1^6^.

We consulted brainspan.org for assessing spatiotemporal gene expression of new FOXP2 target genes in human brain. Sequences of all target genes (new and reproduced ones) and the encoded proteins can be retrieved from the ENSEMBL database via the identifiers (IDs) given in **Table 1**, amongst others. The same IDs lead to the rate ratios of synonymous to non-synonymous substitution rates (dN/dS) of the FOXP2 target genes and genes coding for interactors in the Rhesus monkey–human and Rhesus monkey–common chimpanzee comparison, which we retrieved from the ENSEMBL pages. The sampled dN/dS values were compared with a two-tailed Mann–Whitney *U* (MWU) test as implemented in SPSS (see above). *P*-values from *t*-tests (expressional analyses) were transformed into false discovery rates (FDRs), thus accounting for multiple testing (see, e.g., Vernes et al., 2007). FDRs in GO enrichment analysis as generated by Cytoscape were multiplied by the factor of two, thus conservatively adjusting for parallel testing of two datasets. *P*-values from PPI enrichment testing (network analysis) were adjusted in the same manner. Significance thresholds applied were <0.01 for GO enrichment analysis and <0.05 for all other tests. Data on sample sizes refer to the numbers of cell lines overexpressing either hsa*FOXP2* (N) or different non-human *FOXP2* cDNAs (M).

**Table 1:** Proteins used for network reconstruction and GO enrichment analysis.

Subsample	Symbol	ENSEMBL ID	Subsample	Symbol	ENSEMBL ID
Encoded by reproduced	ADAP1	ENSP00000265846	Added interactors	CFTR	ENSP00000003084
FOXP2 targets	ALG11	ENSP00000430236		DICER1	ENSP00000343745
	APH1A	ENSP00000358105		EIF2C1	ENSP00000362300
	CDH11	ENSP00000268603		EIF2C2	ENSP00000220592
	DNMBP	ENSP00000315659		EIF2C3	ENSP00000362287
	ERP44	ENSP00000262455		EIF2C4	ENSP00000362306
	GPR160	ENSP00000348161		EIF4E	ENSP00000425561
	HSD17B3	ENSP00000364412		EIF4G1	ENSP00000338020
	IFI30	ENSP00000384886		EZR	ENSP00000338934
	IL4R	ENSP00000170630		HSP90AA1	ENSP00000335153
	LONRF1	ENSP00000381298		IL13	ENSP00000304915
	LRP3	ENSP00000253193		IL13RA1	ENSP00000360730
	LRRTM2	ENSP00000274711		IL2RG	ENSP00000363318
	MAFF	ENSP00000345393		IL4	ENSP00000231449
	MARVELD1	ENSP00000441365		JAK1	ENSP00000343204
	MGST2	ENSP00000265498		JAK2	ENSP00000371067
	MRPS6	ENSP00000382250		JAK3	ENSP00000391676
	NEU1	ENSP00000364782		KEAP1	ENSP00000171111
	PCDHB16	ENSP00000354293		KIF13B	ENSP00000427900
	PIM1	ENSP00000362608		LRRK2	ENSP00000298910
	SEMA6D	ENSP00000324857		MRPS10	ENSP00000053468
	SERPINH1	ENSP00000350894		MRPS16	ENSP00000362036
	SETBP1	ENSP00000282030		MRPS2	ENSP00000241600
	TBX22	ENSP00000362390		MRPS5	ENSP00000272418
	TMEM5	ENSP00000261234		NFATC1	ENSP00000327850
	TNRC6C	ENSP00000336783		NFE2L2	ENSP00000380252
	ZDHHC3	ENSP00000296127		PABPC1	ENSP00000313007
Encoded by new FOXP2	BACE2	ENSP00000332979		PAIP1	ENSP00000302768
targets	CDH4	ENSP00000353656		PAN3	ENSP00000370345
	DCDC2	ENSP00000367715		RHOA	ENSP00000400175
	FOXL1	ENSP00000326272		ROCK1	ENSP00000382697
	GABRE	ENSP00000359353		SLC9A3R1	ENSP00000262613
	MSN	ENSP00000353408		SOCS5	ENSP00000305133
	MYH13	ENSP00000252172		STAT3	ENSP00000264657
	MYH8	ENSP00000384330		STAT5A	ENSP00000341208
	NURR1	ENSP00000344479		STAT5B	ENSP00000293328
	PHOX2B	ENSP00000226382		STAT6	ENSP00000300134
	PTPRQ	ENSP00000266688		TARBP2	ENSP00000266987
	SEBOX	ENSP00000416240		TNRC6A	ENSP00000379144
	TMEM200A	ENSP00000296978		TNRC6B	ENSP00000401946

## Results

### Evaluation of the Study System

**FIGURE 2:**
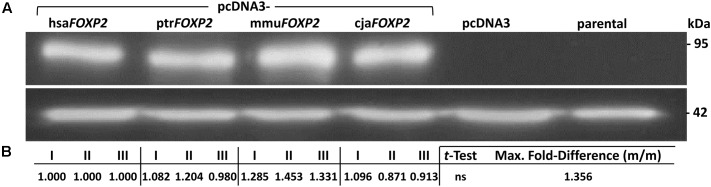
FOXP2 protein expression in SH-SY5Y cells. **(A)** Representative Western blot illustrating that anti-FOXP2 antibody detected no protein in parental cells, indicative of absent endogenous expression. Moreover, there was no FOXP2 detectable in cells carrying empty vector (pcDNA3). In contrast, bands were recognized in SH-SY5Y cells stably transfected with pcDNA3-hsa*FOXP2*, -ptr*FOXP2*, -mmu*FOXP*, and -cja*FOXP2* (all without FLAG tag), thereby indicating similar levels of exogenous FOXP2 expression. Purified lysates were run on an SDS-PAGE gel, transferred to PVDF membrane, and successively hybridized with the respective antibodies. Lower panel: β-actin served as a standard for protein load. **(B)** Densitometric analysis of FOXP2 levels. The given measurements refer to biological replicates I–III (with two technical replicates, each) of the conditions labeled in **(A)**. Maximum (Max.) fold-difference corresponds to the ratio of the most extreme pair of mean expression levels (m/m) between hsa*FOXP2*-overexpressing cells and cells expressing non-human *FOXP2* (here: mmu*FOXP2*). cja, marmoset (*Callithrix jacchus*); hsa, human (*Homo sapiens*); mmu, Rhesus monkey (*Macaca mulatta*); ptr, chimpanzee (*Pan troglodytes*).

RT-PCR detected only minimal endogenous *FOXP2* transcript in parental HEK293 cells (Supplementary Image 1). In further support of their suitability for subsequent validation steps, no FOXP2 band appeared in lanes loaded with lysate from parental HEK293 cells (Western blotting). In contrast, anti-FOXP2 antibody recognized protein bands of two different molecular weights in transiently transfected HEK293 cells, which overexpressed either human or one of the non-human primate *FOXP2* cDNAs. These differences correlated with the extension and non-extension of the different FOXP2 sequences with a C-terminal FLAG tag, thus indicating full length transcription and translation of exogenously expressed *FOXP2* (Supplementary Image 2). After having shown the functionality of the pcDNA3-*FOXP2* constructs we turned to our actual study system, i.e., SH-SY5Y cells. RT-PCR confirmed previous notions of absent endogenous *FOXP2* transcription in parental SH-SY5Y cells (Supplementary Image 1; see also, e.g., Zhao et al., 2015). Consistently, no FOXP2 protein was contained in lysates prepared from parental and pcDNA3-transfected SH-SY5Y cells, according to Western blotting (**Figure 2**). Thus, detection of *FOXP2*/FOXP2 in cells stably transfected with pcDNA3-*FOXP2* constructs can be assigned to exogenous expression. Thereby, we ensured by Sanger sequencing that the different transfectants overexpressed the intended human, chimpanzee, Rhesus monkey, and marmoset FOXP2 cDNA, respectively (not shown). Notably, densitometric analysis suggested about equal FOXP2 protein amounts in hsa*FOXP2*-overexpressing cells on the one hand and SH-SY5Y cells transfected with ptr*FOXP2*, mmu*FOXP2*, and cja*FOXP2* cDNAs on the other (**Figure 2**). Consequently, downstream analyses of target gene expression levels should not be biased by unequal FOXP2 amounts across the cell lines compared.

### Matching of RNA-seq Data with Results of Previous Studies

Preliminary analysis of BPKM values from present RNA-seq (GSE100291) revealed differential expression levels of altogether 898 genes in hsa*FOXP2*-overexpressing SH-SY5Y cells relative to the cells alternatively transfected with ptr*FOXP2*, mmu*FOXP2*, cja*FOXP2* cDNAs, and empty vector (biological replicates I and II per each condition). As to be expected from upstream experiments *FOXP2* was not amongst these differentially regulated genes. However, the sample contained 122 genes that were previously reported to be potential FOXP2/Foxp2 targets (Spiteri et al., 2007; Vernes et al., 2007, 2011; Enard et al., 2009; Konopka et al., 2009) and/or to have singing-related expression regulation in male zebra finch brain (Hilliard et al., 2012). In 27 out of these 122 reproduced genes support for differential BPKM levels under hsa*FOXP2* control was significant according to FDRs <0.05 in the *t*-tests conducted (Supplementary Table 2.1). The corresponding *r* values indicated a large effect size (>0.5) for all 27 comparisons. Consequently, the test power estimates by G^∗^Power constantly overshot the threshold of acceptability, i.e., 80% (Supplementary Table 2.1). The detailed power estimates even ranged from 94 to 100% between the cells overexpressing hsa*FOXP2* (*N* = 2) and non-human *FOXP2* (*M* = 6). This fact, along with the appearance of these 27 genes in the reference studies, suggested that the inclusion of additional replicates should not alter the results. Consequently, we retained all 27 reproduced genes for downstream analyses (Supplementary Table 2.1 and **Table 1**).

### New FOXP2 Targets and Validation by RT-qPCR

Subsequently, we addressed the question if the significance testing of RNA-seq data might have led to an underestimation of the extent of the (hsa)FOXP2-driven network. In order to get an estimate, we selected 13 additional genes out of the aforementioned preliminary set of 898 loci (Supplementary Table 2.2). The genes under scrutiny had low to moderate expression levels but at the same time showed more than twofold differential expression under hsa*FOXP2* control relative to any other condition (mean *versus* mean). Furthermore, they were protein-coding and displayed expression in brain in at least some phase of human life^7^. The 13 genes selected did not receive significant statistical support for FOXP2/Foxp2-driven and/or songbird song-related expression regulation in any of the six reference studies mentioned in the previous paragraph. Thus, we herein refer to the respective genes as to new FOXP2 targets.

RT-qPCR confirmed up- (1 gene) and down-regulation (12 genes) of expression under hsa*FOXP2* control in SH-SY5Y cells for all 13 genes measured (**Table 2**). The effect of hsa*FOXP2* overexpression on target gene expression was again large, as indicated by *r*-values >0.5 in all of the comparisons between hsa*FOXP2* and non-human *FOXP2*-overexpressing cells (biological replicates I and II per each condition). In twelve cases, RT-qPCR corroborated the results of RNA-seq of at least twofold up- or down-regulation (mean *versus* mean) under hsa*FOXP2* control relative to any other condition. We noticed the strongest regulation in *SEBOX*, whereby the minimum fold-change between hsa*FOXP2* and non-human primate *FOXP2*-overexpressing cells was >25 (with *N* = 2, *M* = 6), indicating a strong up-regulation of transcription in response to hsa*FOXP2* overexpression (**Table 2**). The corresponding values for *BACE2*, *CDH4*, *FOXL1*, *GABRE*, *MSN*, *MYH8*, *MYH13*, *NURR1*, *PHOX2B*, *PTPRQ*, and *TMEM200A* ranged between 0.037 and 0.397, which corresponds to a considerable down-regulation of transcription for each of these loci. *DCDC2* failed the twofold-threshold in the comparison of the hsa*FOXP2*-overexpressing cells with any other condition (0.572) but down-regulation of expression under hsa*FOXP2* control was nonetheless significant (**Table 2**). Significant FDRs (<0.05, *t*-test) were also reached in the human/non-human comparison of the other new FOXP2 target candidates, except for *NURR1* and *TMEM200A* (all with *N* = 2 and *M* = 6). The latter two genes missed the 5% threshold of significance in the first place, despite their strong down-regulation in hsa*FOXP2*-overexpressing cells and the correspondingly increased *r*-values (>0.7).

**Table 2:** Target gene expression levels (RT-qPCR) in SH-SY5Y cells overexpressing human *FOXP2* relative to cells overexpressing non-human primate *FOXP2*.

	pcDNA3-											
	hsa*FOXP2*		ptr*FOXP2*		mu*FOXP2*		cja*FOXP2*					
Symbol	I	II	I	II	I	II	I	II	FDR *t*-test	Min. fold-change (m/m)	*r*	Power
*BACE2*	0.177	0.211	1.875	1.973	1.598	1.999	0.595	0.497	<0.001	0.355	0.735	0.725
	0.177	0.211	1.875	1.973	1.598	1.999	–	–	<0.05	0.108	0.986	1.000
*DCDC2*	0.289	0.230	0.562	0.879	0.433	0.474	0.889	0.800	<0.01	0.572	0.767	0.803
*CDH4*	0.284	0.387	1.128	1.661	3.003	3.565	2.437	2.154	<0.05	0.241	0.808	0.895
*FOXL1*	0.378	0.308	3.404	2.443	3.140	3.661	2.235	1.790	<0.001	0.170	0.897	0.997
*GABRE*	0.014	0.012	0.423	0.455	0.606	0.562	0.361	0.348	<0.01	0.037	0.933	1.000
*MSN*	0.111	0.180	1.217	1.825	0.981	1.237	1.391	1.474	<0.01	0.131	0.931	1.000
*MYH8*	0.253	0.293	1.369	1.620	1.337	1.285	1.974	2.067	<0.01	0.208	0.923	1.000
*MYH13*	0.044	0.049	1.011	0.897	0.565	0.536	0.808	0.826	<0.01	0.084	0.922	1.000
*NURR1*	0.092	0.131	2.742	2.331	3.386	3.915	0.840	1.007	ns	0.121	0.743	0.745
	0.092	0.131	2.742	2.331	3.386	3.915	–	–	0.01	0.044	0.943	0.999
*PHOX2B*	0.091	0.083	0.942	0.598	0.877	0.447	2.598	3.094	<0.05	0.131	0.589	0.411
	0.091	0.083	0.942	0.598	0.877	0.447	–	–	<0.05	0.131	0.873	0.899
*PTPRQ*	0.147	0.131	0.401	0.300	1.357	1.119	0.331	0.377	<0.05	0.397	0.556	0.357
	0.147	0.131	0.401	0.300	–	–	0.331	0.377	<0.01	0.397	0.950	1.000
*SEBOX*	4.423	4.172	0.105	0.143	0.143	0.169	0.153	0.182	<0.05	25.657	0.998	1.000
*TMEM200A*	0.124	0.088	1.110	0.839	1.567	1.207	0.326	0.483	ns	0.262	0.731	0.715
	0.124	0.088	1.110	0.839	1.567	1.207	–	–	<0.05	0.109	0.921	0.987

*Two biological replicates (I, II) were measured per condition. Values were corrected for signal intensity of reference genes and normalized for expression levels in cells carrying empty expression vector. Minimum (Min.) fold-difference refers to the ratio of the least extreme pair of mean expression levels between hsa*FOXP2*-overexpressing cell lines and their counterparts overexpressing one of the non-human *FOXP2* cDNA. Power estimates according to G^∗^Power v. 3.1.9.2. FDR, false discovery rate; cja, marmoset (*Callithrix jacchus*); hsa, human (*Homo sapiens*); mmu, Rhesus monkey (*Macaca mulatta*); ns, not significant; ptr, chimpanzee (*Pan troglodytes*); *r*, correlation coefficient*.

This discrepancy between effect size and significance testing in *NURR1* and *TMEM200A* apparently reflected an increased variation across the non-human models due to conspicuous values under cja*FOXP2* control. However, the inclusion of models for the Rhesus monkey and marmoset besides the chimpanzee condition was a rather conservative approach with respect to our prime goal of detecting expressional changes in the human model cell lines (compare **Figure 1B**). In *NURR1* and *TMEM200A*, the consideration of the complete species sample might even have obscured actually relevant changes in response to hsa*FOXP2* overexpression. Accordingly, we found the expression levels of *NURR1* and *TMEM200A* to differ significantly under hsa*FOXP2* control when the data were re-analyzed under exclusion of the values measured in the cja*FOXP2*-overexpressing cells (thus, with *N* = 2 and *M* = 4; **Table 2**). The reduction in the species sample associated with an increase of the corresponding *r* values and power estimates for the *t*-tests carried out on *NURR1* and *TMEM200A*. Similarly, the power estimates overshot the 80% threshold of acceptability when the levels of *BACE2*, *PHOX2B*, and *PTPRQ* transcripts were compared between hsa*FOXP2*-overexpressing cells and a reduced sample of non-human primate models (**Table 2**). Thus, *post hoc* analysis of *t*-tests underlined that significant support for expressional changes under hsa*FOXP2* control could be correlated with acceptable power estimates in all 13 new target genes – at least after obscuring signal was excluded from the comparison. However, high power estimates suggest that the alternative hypothesis of unequal means (here: expression levels) is true. Consequently, additional biological replicates should reproduce the findings without bringing an essential gain of new information – a prediction that we tested on the protein level.

### Western Blotting of Proteins Encoded by New FOXP2 Targets

**FIGURE 3:**
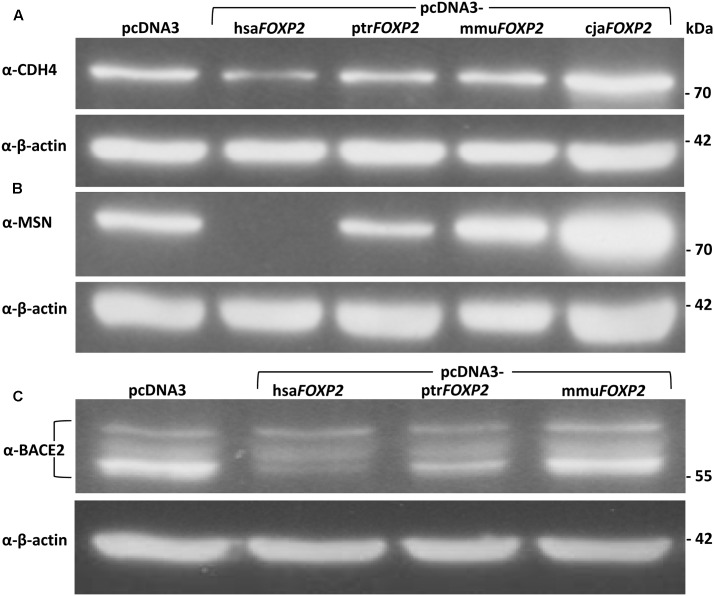
Representative Western blots showing expression levels of FOXP2 targets in SH-SY5Y cells. **(A)** Anti-CDH4 detected significantly less protein quantity in response to hsa*FOXP2* overexpression relative to the cells overexpressing one of the three non-human primate *FOXP2* cDNAs (all without FLAG tag). **(B)** No MSN was discernible in hsa*FOXP2*-overexpressing cell lines, whereas protein expression was obvious in cell lines either overexpressing one of the tested non-human primate *FOXP2* cDNAs or carrying empty vector (pcDNA3). **(C)** Anti-BACE2 recognized one band at about 56 kDa and additional ones at higher molecular weights, which presumably represent differently glycosylated variants of the membrane protein (Acquati et al., 2000). In particular, the lowest band appeared to be down-regulated under hsa*FOXP2* control relative to the non-human models included, but all the bands were used for densitometric analysis. Purified lysates were run on an SDS-PAGE gel, transferred to PVDF membrane, and successively hybridized with the respective antibodies. Lower panels: β-actin served as a standard for protein load in all experiments. Results of densitometric analyses are given in **Table 3**. cja, marmoset (*Callithrix jacchus*); hsa, human (*Homo sapiens*); mmu, Rhesus monkey (*Macaca mulatta*); ptr, chimpanzee (*Pan troglodytes*).

The three proteins selected for Western blot analyses represented loci, for which the power of *t*-tests was >80% when contrasting transcript amounts (RT-qPCR) in hsa*FOXP2*-overexpressing SH-SY5Y cells with the corresponding levels in either the complete (*CDH4*, *MSN*) or a reduced set of non-human models (*BACE2*). Densitometric analysis confirmed significant down-regulation under hsa*FOXP2* control for all three tested proteins, when taking the same two biological replicates per condition as used for transcriptome measurements (I and II). As in transcriptomic analyses, the *r* values exceeded the threshold of large effect size in all of the three comparisons of densitometric values (**Figure 3** and **Table 3**). Correspondingly, the power estimates for the conducted *t*-tests was constantly >80%, so that the inclusion of additional replicates should not alter the results. In line with this expectation, *t*-tests confirmed a significant down-regulation of protein expression after addition of a third biological replicate (III), thus increasing sample sizes to *N* = 3 and *M* = 9 for CDH4 and MSN, and to *N* = 3 and *M* = 6 for BACE2 (**Figure 3** and **Table 3**). We interpreted these findings as a confirmation that SH-SY5Y cells translated different transcript amounts of FOXP2 targets into corresponding protein quantities. The findings further demonstrated that large effect size and high power values appeared to be reliable predictors of the reproducibility of *t*-test results, even when sample sizes were comparably small. In retrospect, therefore, the sample sizes in the transcriptome analyses seemed acceptable.

**Table 3:** Densitometric analyses of Western blots: CDH4, MSN, and BACE2 abundance in SH-SY5Y cells overexpressing human *FOXP2* relative to cells overexpressing non-human primate *FOXP2*.

	Overexpression of														
	hsa*FOXP2*			ptr*FOXP2*			mmu*FOXP2*			cja*FOXP2*					
Protein	I	II	III	I	II	III	I	II	III	I	II	III	FDR *t*-test	*r*	Power
CDH4	0.431	0.650	–	0.952	1.171	–	1.018	1.045	–	1.336	1.213	–	<0.01	0.861	0.976
	0.431	0.650	0.514	0.952	1.171	0.960	1.018	1.045	2.499	1.336	1.213	2.691	<0.05	–	–
MSN	0.051	0.116	–	0.695	0.728	–	2.046	1.694	–	2.562	2.228	–	<0.05	0.775	0.822
	0.051	0.116	0.077	0.695	0.728	0.595	2.046	1.694	1.759	2.562	2.228	2.204	<0.001	–	–
BACE2	0.358	0.348	–	0.749	0.731	–	1.071	0.989	–	–	–	–	<0.001	0.900	0.958
	0.358	0.348	0.344	0.749	0.731	0.974	1.071	0.989	1.164	–	–	–	<0.05	–	–

*Two and three biological replicates per condition (I–III) were included in *t*-tests. Values were corrected for β-actin levels and normalized for protein levels of the respective protein in cells carrying empty vector. See **Figure 3** for representative Western blots. cja, marmoset (*Callithrix jacchus*); hsa, human (*Homo sapiens*); mmu, Rhesus monkey (*Macaca mulatta*); ptr, chimpanzee (*Pan troglodytes*)*.

### Mapping of FOXP2-Binding Motifs and FOXP2-ChIP-seq Reads to Putative Promoter Sequences of New FOXP2 Targets

**FIGURE 4:**
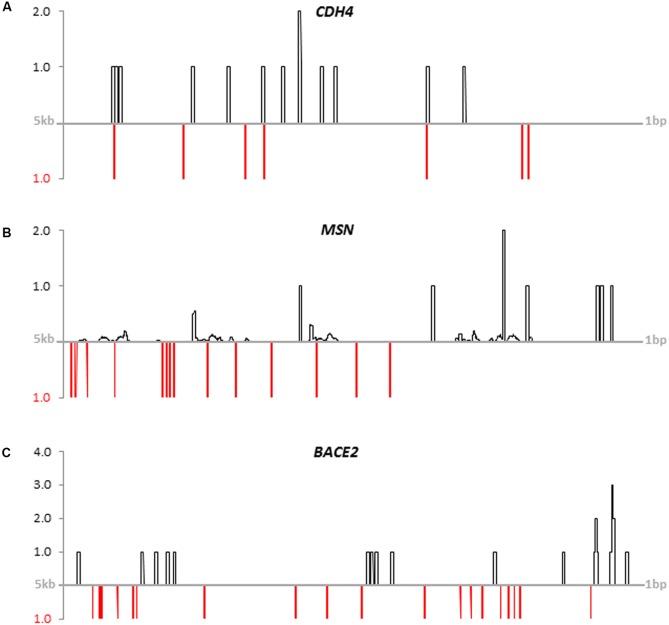
Coverage of publicly available FOXP2-ChIP-seq reads (black lines) and FOXP2-binding motifs (red lines) on putative promoter sequences. **(A)** *CDH4*, **(B)** *MSN*, and **(C)** *BACE2*. Putative promoter sequences of human genes spanned 5 kb upstream of the transcription start sites. FOXP2-ChIP-seq reads were down-loaded from NCBI’s GEO database (GSM803353: SRR351544). Hits were normalized for the genome-wide background (GRCh38.p7). See Supplementary Table 2.3 for sequence IDs and motifs. Mapping results for the other eleven new FOXP2 targets are given in Supplementary Image 3.

After having shown differential expression under hsa*FOXP2* control for all 13 new candidate genes we investigated their regulatory sequences. Screening the 5 kb upstream the transcription start of the human orthologs we found numerous matches with publicly available FOXP2-ChIP-seq reads (SRR351544). The same putative promoter sequences additionally contained previously published FOXP2-binding motifs (see Stroud et al., 2006; Vernes et al., 2007; Nelson et al., 2013). The number of matches further increased when extra motifs overrepresented in murine Foxp2 target gene promoters were taken into account (see Vernes et al., 2011). Overall, we detected between seven and 48 FOXP2/Foxp2-binding motifs within the putative human promoter sequences (Supplementary Table 2.3). Furthermore, we observed juxtaposition and overlaps of motifs and the matching positions of FOXP2-ChIP-seq reads in the promoter sequences of all 13 new target genes (see **Figure 4** for *CDH4*, *MSN*, and *BACE2*; see Supplementary Image 3 for the other genes). Thus, the expression of all 13 new candidate genes might be directly regulated by FOXP2. Whether in terms of a direct or indirect regulation through FOXP2 we accepted all 13 genes scrutinized for downstream network reconstruction.

### Network Reconstruction, Gene Ontology Enrichment Analysis, and Evolutionary Analysis

**FIGURE 5:**
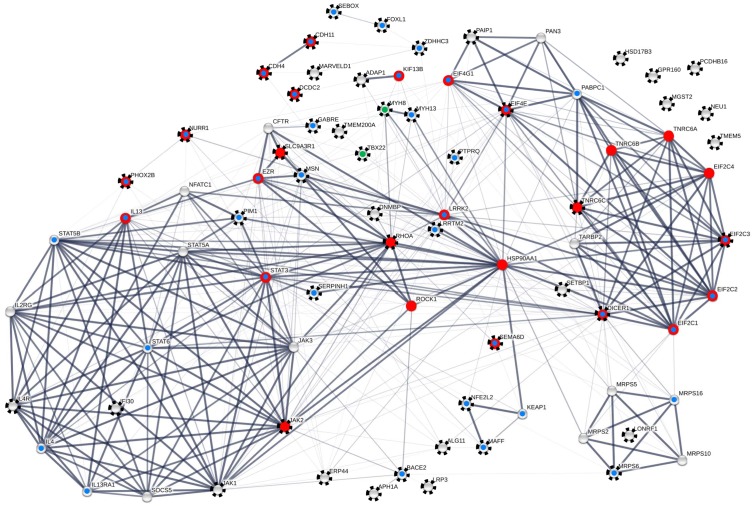
FOXP2-driven protein–protein interaction (PPI) network including 80 proteins. Seventy-four nodes are contained in the largest connected component (LCC). Black rays highlight 49 proteins with empirical evidence for FOXP2/Foxp2-driven and/or songbird-song-related expression regulation from the present and previous analyses (see **Tables 2**, **3** as well as Supplementary Tables 2.1, 2.4 for details). Red dots highlight proteins that matched with neuron-related GO terms in enrichment analysis (see Supplementary Table 2.8). Blue dots indicate nodes whose implication in neuronal and neural functions and/or diseases and disorders is detailed in the Discussion. This is a conservative estimate as exemplified by ERP44 which might have neural relevance (see Discussion) but is not categorized as such in this scheme. Green dots refer to an involvement in trismus-pseudocamptodactyly (MYH8) and X-linked cleft palate and ankyloglossia (TBX22). Proteins in the LCC have 11.342 direct interactors (node degree) on average. Thickness of edges correlates with confidence scores ≥0.90, ≥0.7, ≥0.4, and ≥0.15. The clustering coefficient varied between 0.616 and 0.831 depending on the confidence threshold applied. The network was constructed with the aid of STRING v. 10.0 and analyzed with the aid of Cytoscape v. 3.2.1 and the NetworkAnalyzer plugin. For individual node degrees and additional network statistics see Supplementary Tables 2.6, 2.7.

For network reconstruction, we merged our 13 new with the 27 reproduced target genes, thus generating an initial sample of 40 genes with empirical evidence for FOXP2/Foxp2-driven expression regulation. In order to reach a meaningful size for network and GO analyses, the STRING server was enabled to add best-supported 40 interactors so that the final dataset contained 80 proteins (**Table 1** and Supplementary Tables 2.4, 2.5). Interestingly, these interactors contained nine additional proteins whose coding genes were previously shown to be FOXP2/Foxp2 targets and/or to associate with singing in male zebra finch brains (Supplementary Table 2.4). Thus, altogether 49 nodes in the network correlated with empirical support for FOXP2/Foxp2-driven and/or songbird song-related expression regulation (nodes with rays in **Figure 5**). Disregarding six genes with only songbird song-related expression regulation a total of 43 nodes in our network associated with experimental evidence for FOXP2/Foxp2-driven expression regulation (**Table 1** and Supplementary Tables 2.1, 2.4). For this reason, we further refer to the network as to the FOXP2-driven network (**Figure 5**).

The number of PPIs was significantly increased relative to the expectation whichever confidence threshold was applied (*FDR* = 0, each; Supplementary Tables 2.6, 2.7). Fifty-three percent of the interactions were recognized with at least medium confidence (≥0.4). Confidence was still high (≥0.7) in 43% and highest (≥0.9) in 36% of the edges (**Figure 5** and Supplementary Table 2.7). Self-interactions were not detected. Six of the proteins were not engaged in any interaction. The remaining 74 proteins were constituents of the largest connected component (LCC), thereby having 11.342 PPI partners on average (see **Figure 5**). With 36 PPIs, the chaperone HSP90AA1 was the most connected protein in the LCC. Out of the new FOXP2 targets, MSN was the only one with an above-average node degree (= 15; **Figure 5** and Supplementary Table 2.6).

**FIGURE 6:**
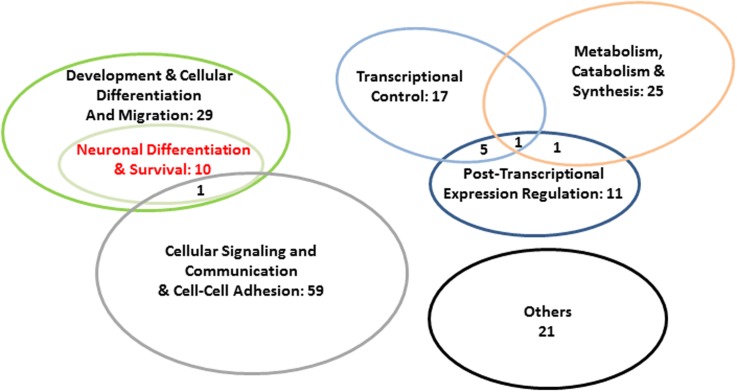
Abundance of super-ordinate categories of the enriched biological process GOs in our 80 protein sample. GO enrichment analysis was carried out with STRING v. 10.0 (FDR < 0.01). For detailed results of GO analysis, see Supplementary Table 2.8.

Kinases with high node degrees such as LRRK2 and Janus kinases JAK1-3 but also above-average connected PIM1 and average-connected ROCK1 pointed to a general involvement of our network in the regulation of protein activity. The same is true for ROCK1’s highly connected upstream regulator RHOA, and for the phosphatase PTPRQ. Also the regulation of conductivity was represented by our network, namely through CFTR and GABRE (compare **Figure 5**; see Discussion for detailed protein functions). Additional functional implications emerged when testing our 80 protein sample for the enrichment of GO terms (STRING). Applying a 1% FDR, 153 biological process GOs were overrepresented in our sample relative to the genome-wide background (**Figure 5** and Supplementary Table 2.8). When individual terms were combined to larger entities, cellular signaling and communication appeared as the largest category (number of GO terms = 59; **Figure 6** and Supplementary Table 2.8). This category contained members of the JAK/STAT cascade (statins, Janus kinases, and SOCS5) as well as MSN and EZR as constituents of the ezrin–radixin–moesin complex (ERM). Also cell–cell adhesion-mediating cadherins (CDH4, CDH11) were sorted into this category. Twenty-five GOs demonstrated importance for metabolic and catabolic processes as illustrated by ZDDH3, KEAP1, and LRRK2. Seventeen GOs reflected a strong involvement in transcriptional control (**Figure 6**). The latter category included transcriptionally active proteins like MAFF, NFATC1, and TBX22 as well as proteins encoded by the new FOXP2 targets *NURR1*, *PHOX2B*, *FOXL1*, and *SEBOX*. High relevance for post-transcriptional expression regulation was suggested by altogether eleven respective GOs. Proteins acting in ribosome recruitment (EIF4E, EIF4G1, and PABPC1) fell under this category. The same applied to highly connected DICER1, TARBP2, TRNC6A-C, and four RISC members (EIF2C1-4) which conjointly function in gene silencing. Actually, terms relating to gene silencing received the highest support from GO enrichment analysis (Supplementary Table 2.8). Five mitochondrial ribosomal proteins pointed to an engagement in protein synthesis as an additional layer of post-transcriptional expression regulation. Twenty-nine GOs indicated increased pertinence for development and cellular differentiation, migration, and motility. Notably, 10 out of these 29 GOs were directly relating to nervous system development and to neuron differentiation, projection, morphogenesis (inclusively axonogenesis and neurotrophin signaling) and survival (**Figure 6**). The 26 proteins matching these “nervous system terms” are highlighted by red dots in **Figure 5** (compare Supplementary Table 2.8).

For assessing the impact of the newly detected FOXP2 targets we repeated network and GO enrichment analyses under exclusion of the respective 13 proteins, thus starting with the 27 reproduced loci only. After addition of 40 interactors STRING again detected significantly more nodes than expected, no matter which confidence threshold was applied (Supplementary Tables 3.1, 3.2). About 82% of the previously recognized enriched GO terms were also reproduced. However, with the exception of a single GO term (neurotrophin TRK receptor signaling pathway) there was no enrichment of terms literally relating to neuronal relevance anymore. In particular, terms containing the words “neuron” or “axonogenesis” were not enriched in the 67 protein sample (Supplementary Table 3.3). Thus, the inclusion of the new FOXP2 targets significantly affected the results of GO enrichment analysis.

Lastly, we analyzed the sequence evolution of the 80 protein sample on the basis of 59 protein-coding genes for which the respective values were available at the time of the study (ENSEMBL). Taking dN/dS as a measure we found overall similar evolutionary rates in the Rhesus monkey–human and Rhesus monkey–chimpanzee comparison. This was evidenced at the level of mean dN/dS values (0.202 *versus* 0.200, respectively) as well as medians (0.148 *versus* 0.141, respectively; *P* = 0.957, MWU test). The single dN/dS values were all <1.0 (Supplementary Table 2.9), thus illustrating that negative selection and hence selection against aa exchanges prevailed in the evolution of the present FOXP2-driven network.

## Discussion

Comparing human neuronal cells (SH-SY5Y) stably overexpressing species-specific primate *FOXP2* cDNAs, we detected 40 genes with differential expression levels in response to hsa*FOXP2* overexpression. We refer to 27 of these genes as to reproduced FOXP2 targets as they have been already reported to show FOXP2/Foxp2-driven and/or songbird sing-related expression regulation in several reference studies (Spiteri et al., 2007; Vernes et al., 2007, 2011; Enard et al., 2009; Konopka et al., 2009; Hilliard et al., 2012). The remaining 13 genes with differential expression levels controlled by hsa*FOXP2* did not show significantly differential regulation in any of the references and hence are termed new FOXP2 targets herein.

In support of the validity of the findings, *post hoc* analyses revealed that the recognition of transcriptional changes under hsa*FOXP2* control associated with acceptable power (>80%) of the conducted *t*-tests. This might be surprising on the first sight, considering that mRNA-based measurements involved comparably few cell lines modeling the human and non-human conditions, with a total sample size of mostly 8 and exceptionally 6 (**Table 2** and Supplementary Table 2.1). However, such high power estimates fully agree with the results of a simulation study which demonstrated that *t*-tests can reach acceptable power despite small sample sizes (*N* = 2; *M* = 5; thus, with a total sample size of 7), when the corresponding effect sizes are high (de Winter, 2013). The precondition of large effect size was indeed fulfilled in present transcriptome analyses (**Table 2** and Supplementary Table 2.1). As high power estimates suggest that the alternative hypothesis of unequal means is true, the inclusion of additional biological replicates should not have altered the results in present transcriptome analyses (see Cohen, 1988; Open Science Collaboration, 2012) – an assessment which we found confirmed for a selection of proteins encoded by new FOXP2 targets (**Table 3**). Therefore, it seemed justifiable to us to take all 40 genes which showed differential transcription under hsa*FOXP2* control as a starting sample for network reconstruction and evolutionary analysis.

The resulting FOXP2-driven network contained altogether 80 proteins. Matching the newly added interactors with the reference studies increased the number of nodes with FOXP2/Foxp2-driven and/or songbird song-related expression regulation in the network to a total of 49 (nodes with rays in **Figure 5**). Forty-three of them correlated with experimental evidence for FOXP2/Foxp2-driven expression regulation from the present and several reference studies (see Results). Considering FOXP2’s role in impairment of verbal communication (see Introduction), our FOXP2-driven network might have played a contributory role in human evolution, potentially even in the acquisition of speech and language (**Figures 1**, **5**). However, adaptive aa substitutions were apparently of minor importance in this context as illustrated by prevalent signatures of negative selection, i.e., selection disfavoring aa exchanges, in genes coding for the proteins in our network (Supplementary Table 2.9). The codons of the respective genes might even evolve under stronger constraint than it is the case across the entire genome. Thus, the mean dN/dS of the genes encoding our network members was 0.202 in the Rhesus monkey-human comparison, while the genome-wide mean should be in the range of 0.26 or higher according to the dN and dS values, which Wolf et al. (2009) reported for the same species pair. This could point to an increased functional relevance of the FOXP2-driven network in primate evolution. If true, this seems to be a general principle as we observed similar evolutionary rates of the genes encoding our network members, whether the Rhesus monkey orthologs were compared with their counterparts in humans or common chimpanzee (Supplementary Table 2.9). On the contrary, our data do not suggest noteworthy changes in the evolutionary rates of the members of the FOXP2-driven network on the human branch (compare **Figure 1B**). This does not change the fact that adaptive evolution of some genes influenced hominization as it is the case for FOXP2 itself (Mallick et al., 2016; also, e.g., Enard et al., 2002; Mozzi et al., 2016). Still, present results of RNA-seq, RT-qPCR, and Western blotting rather emphasize the prominent role of expression regulation changes in human evolution (**Tables 2**, **3** and Supplementary Tables 2.1, 2.2), thus lending support to respective postulates from about 30 years ago (e.g., King and Wilson, 1975). The changes in fine-tuning might have affected cellular signaling and communication, protein and nucleotide metabolism and catabolism, expression regulation, development and cellular differentiation and migration, and especially neuronal differentiation and survival (**Figure 6**). For reasons of space limitations we will focus in the following on the respective implications of the LCC in the present FOXP2-driven PPI network (**Figure 5**).

### Cytoskeleton: MSN, the ERM Complex, and the Actin Scaffold

Above-average connected moesin (MSN, also MOE) was expressed at markedly lower levels in SH-SY5Y cells stably transfected with hsa*FOXP2* relative to cells overexpressing non-human primate *FOXP2* cDNAs (**Figures 3B**, **5** and **Tables 2**, **3**). In support of its neuronal relevance, MSN protein levels were previously reported to be down-regulated in fetal Down syndrome brains (Lubec et al., 2001) whereas levels of a *MSN*-binding non-coding RNA (*MSNP1AS*) showed up-regulation in ASD cortices (Kerin et al., 2012). Such associations might reflect the central role of the protein in the remodeling of the cell cortex during mitosis and also its activation by the phosphatase PTEN (see Roubinet et al., 2011; also Georgescu et al., 2014). Hence, PTEN is a critical regulator of neuron development and survival, axonal regeneration, and synaptic plasticity and is implicated in AD, PD, and ALS (Ismail et al., 2012). Recent observations in the mouse model fit in with the presumed functional association of the three proteins. Thus, mislocalization of Pten in murine brain was observed to correlate with down-regulation of *Foxp2* and upregulation of *Msn* (Tilot et al., 2016).

Although MSN seems to function on its own (e.g., Fehon et al., 2010; Roubinet et al., 2011), it is also active through its participation in the ERM complex which additionally contains RDX and the present LCC member EZR (**Figure 5**). The ERM complex bridges the plasma membrane with the actin cytoskeleton, thus being involved in cell–cell recognition, signaling, and motility of diverse cell types as well as the formation and collapse of filopodia, microvilli, and microspikes (e.g., Fehon et al., 2010; Antoine-Bertrand et al., 2011; Roubinet et al., 2011; Georgescu et al., 2014). Accordingly, MSN and EZR matched in the present enrichment analysis with more general GO terms such as movement of cell or subcellular component and membrane to membrane docking (Supplementary Table 2.8). Nonetheless, the functional spectrum of the ERM complex also covers regulation of neurite outgrowth, neuron motility and growth cone morphology (e.g., Antoine-Bertrand et al., 2011 and references therein). These functions obviously substantiate the neuronal relevance of the present FOXP2-driven network – and of its LCC.

Activation of the complex through phosphorylation (pERM) involves three additional members of our LCC (**Figure 5**), i.e., RHOA, RHOA’s downstream effector and regulator ROCK1 (Antoine-Bertrand et al., 2011; Tang et al., 2012), and LRRK2 (Parisiadou et al., 2009). ROCK1 is implicated in neuronal regeneration and neuritogenesis (Da Silva et al., 2003; Tang et al., 2012). Moreover, mutations in *LRRK2* gene represent the most frequent genetic cause of late-onset PD (Paisan-Ruiz et al., 2008), possibly due to negative effects on neuritogenesis and survival of nigrostriatal dopaminergic neurons (Han et al., 2008; Xiao et al., 2015). The links between our LCC and neurodegeneration are even more manifest when considering that pERM is required for proteolytic processing of amyloid precursor protein (APP) by α-secretases into the neuroprotective soluble APP ectodomain (sAPPα) (Darmellah et al., 2012). Yet, the alternative cleavage of APP into neurotoxic amyloid-β (Aβ) is catalyzed by a γ-secretase containing present LCC member APH1A (Zhao et al., 2010) (**Figure 5**). Another APP processing pathway involves the aspartic protease encoded by the new FOXP2 target *BACE2* (β-site APP-cleaving enzyme 2; also *CEAP1*, *DRAP*) which resides inside the so-called ‘Down critical region’ in 21q22.3 (Acquati et al., 2000; O’Brien and Wong, 2011) (**Figures 3C**, **5** and **Table 2**). In line with the expectation for BACE2’s ability to cleave APP, certain variants of the coding gene associate with neurodegeneration, namely with AD (Myllykangas et al., 2005). However, the connections of our LCC with APP metabolism are not confined to the cleaving enzymes. Thus, the present LCC also contains the chaperone SERPINH1 (also HSP47) which has been demonstrated to regulate Aβ formation and the growth of amyloid plaques (**Figure 5**) (Bianchi et al., 2011).

### Cytoskeleton: Myosins and Microtubules

The two myosin heavy chain proteins in the present LCC, both encoded by new FOXP2 targets (**Figure 5** and **Table 2**), might have an influence on hard tissue development. Thus, *MYH8* levels were found to be up- and down-regulated in retrognathia and prognathia patients, respectively (Oukhai et al., 2011). Furthermore, a recurrent mutation in *MYH8* gene associates with trismus-pseudocamptodactyly syndrome (TPS) involving joint contracture and the inability of patients to open the mouth fully (also Dutch-Kentucky or Hecht-Beal syndrome; e.g., Toydemir et al., 2006). Strikingly, a recent study demonstrated the expression of *Foxp2* (and *Foxp1*) in the developing temporomandibular joint of mice (Cesario et al., 2016). Consequently, disturbed FOXP2-regulated expression of *MYH8* might indeed play a role in the pathogenesis of TPS. Also MYH13 seems to be important for the development of the anatomical basis of speaking. The protein might especially be involved in the acquisition of adult larynx properties as suggested by cease of laryngeal MYH13 expression during or after childhood (Périé et al., 2000). *MYH13* seems further to be involved in the pathogenesis of age-related neurodegenerative disorders (e.g., Cacabelos et al., 2012) and in formal thought disorder, or disorganized speech (Wang et al., 2012).

Besides, our FOXP2-driven network contributes to the organization of the microtubule scaffold. In particular, the new FOXP2 target *DCDC2* codes for a protein (**Figure 5** and **Table 2**) which directs neuronal migration by stabilizing microtubules (e.g., Meng et al., 2005). This functional implication might have importance for human communication skills as suggested by mutations in *DCDC2* that associate with a recessive form of deafness (DFNB66), variation of gray matter volume in language-related brain regions of schizophrenia patients, reading disability (RD), and dyslexia (DYX2) (Meng et al., 2005; Jamadar et al., 2011; Newbury et al., 2011; Grati et al., 2015). In line with these associations in humans, *DCDC2* is co-expressed in certain regions of the marmoset brain with other speech- and language-related genes like *FOXP2* itself, but also with *ROBO1*, *CMIP*, *KIAA03319*, and *CNTNAP2*. The spatiotemporal overlap includes thalamus and basal ganglia and, especially, substantia nigra pars compacta and pars reticulata (Kato et al., 2014; see also Vernes et al., 2008). Yet, nigrostriatal and thalamocortical-basal ganglia circuits function in voluntary motor control in marmoset (Kato et al., 2014) and dysfunction in humans can lead to oromandibular, lingual, and laryngeal spasms (Colosimo et al., 2010). Thus, *DCDC2* and the co-expressed speech- and language genes exemplify that the study of marmoset can improve our understanding of the molecular and neural basis of human communication. At the same time, *DCDC2* exemplifies that the differences in the communication skills between humans and marmoset might be due to changes in expression levels, which occurred on the human branch (compare **Figure 1B**).

Similar to DCDC2, MARVELD1 has a rather peripheral position in our LCC. Nonetheless, also this protein has importance for the organization of microtubules as demonstrated for the murine ortholog (Zeng et al., 2011). Microtubules are further the basis for the functioning of present LCC member KIF13B (**Figure 5**). The protein moves along microtubules to the tips of neurites where it promotes neurite outgrowth (Yoshimura et al., 2010). Notably, the murine protein has been shown to be a negative regulator of PIK3K/AKT-mediated myelination in central and peripheral nervous system (Noseda et al., 2016). Yet, phosphatidylinositol-mediated signaling was one of the enriched GO terms in our protein sample (Supplementary Table 2.8). Moreover, PIK3K/AKT signaling could also link the new FOXP2 target *PTPRQ* (DFNB84A) (**Table 2**). The gene is another deafness susceptibility locus in our LCC (**Figure 5**) and codes for a phosphatidylinositol phosphatase (e.g., Schraders et al., 2010) which has been implicated in the organization of the actin cytoskeleton again (see, e.g., Nayak et al., 2007).

### Transcriptional Regulation: Transcription Factors

A number of proteins in our LCC support FOXP2’s previously stated influence on neuronal development and maintenance through downstream transcriptional regulators (**Figures 5**, **6**). Thereby, the transcription factor encoded by the new FOXP2 target *NURR1* (also *NR4A2*, *NOT*) (**Figure 5** and **Table 2**) seems to be of special importance for normal dopaminergic functioning. Thus, stimulation of NURR1 improves behavioral deficits associated with the degeneration of dopamine neurons in PD model mice – an effect which involves enhanced *trans*-repression of neurotoxic pro-inflammatory genes in microglia and increased transcriptional activation of midbrain dopaminergic (mDA) neurons (Kim et al., 2015). *Nurr1* knockout mice even fail to develop dopamine neurons (e.g., Zetterström et al., 1997). Therefore, it is not surprising that several mutations in human *NURR1* coincide with dopamine-related diseases, namely SCZD, Lewy body dementia (LBD), AD, and PD (e.g., Chen et al., 2001; Zheng et al., 2003; Chu et al., 2006). The involvement of the present LCC in neuronal maintenance is also reflected by MAFF (**Figure 5**), i.e., another transcription factor, which has also been implicated in PD (reviewed in Kannan et al., 2012).

Four additional proteins further substantiate FOXP2’s effectivity through downstream regulators of transcription (**Figures 5**, **6** and see **Tables 2**, **3** for new and reproduced FOXP2 targets). Corresponding evidence is particularly strong with respect to PHOX2B: Murine Phox2b regulates the differentiation of hindbrain visceral and branchial motor neurons (see Hirsch et al., 2013). *Phox2b* knockout mice even lack the facial motor nucleus which is an important source of Slit ligands for Robo receptor-expressing pontine neurons in wild-type mice (Geisen et al., 2008). Yet, the essentiality of SLIT1/ROBO signaling for neuron migration and axon guidance is well established (see, e.g., Geisen et al., 2008; Boeckx and Benítez-Burraco, 2014a,b; Pfenning et al., 2014), and *SLIT1* belongs to the already known FOXP2 targets (Konopka et al., 2009; Devanna et al., 2014). The second protein out of this group of four, SEBOX is involved in postnatal brain maturation as suggested by corresponding evidence in the mouse model (Cinquanta et al., 2000). Seeing the remarkable up-regulation of *SEBOX* under hsa*FOXP2* control (**Table 2**) the encoded protein might indeed have importance for human brain development and evolution. The third one, FOXL1 could play a role in (mid)brain development as suggested by respective observations in the zebrafish (Nakada et al., 2006). Similarly, the functioning of the transcription factor TBX22 has a morphogenetic dimension: Loss-of-function mutations in the coding gene cause X-linked cleft palate and ankyloglossia, a developmental disorder which decreases the motility of the tongue, thus leading to problems with feeding and speech. The disorder also affects dentition, hearing, and psychological development (Braybrook et al., 2001) Thus, the transcriptional cascades controlled by FOXP2 are essential for neural and neuronal maintenance and development as well as for normal development of the anatomical underpinning of speech.

### Transcriptional Regulation: JAK/STAT Signaling

JAK/STAT signaling, represented in the present LCC by highly connected three Janus kinases (JAK1-3) and four statins (STAT3, STAT5A, STAT5B, STAT6) (**Figures 5**, **6**), is commonly connoted with immune reaction (see Supplementary Table 2.8). However, a growing body of data points to a contributory role of the JAK/STAT cascade in the pathogenesis of Down syndrome, neuro-inflammatory diseases, and dopaminergic neurodegeneration (Lee et al., 2016; Qin et al., 2016). In agreement with the symptoms associated with these pathologies, present LCC members STAT3, STAT5B, and STAT6 modulate neuron survival, synaptic plasticity, and neurite outgrowth (Deboy et al., 2006; Georganta et al., 2013; Tyzack et al., 2014).

The JAK/STAT cascade additionally includes interleukins, their receptors, and members of the suppressor of cytokine signaling (SOCS) protein family (also STAT-induced STAT inhibitor family). For example, interaction of Socs5 with Il4r inhibits Il4-dependent activation of Stat6 in the mouse model (Seki et al., 2002), and expression of Il4 can again be induced by Nfatc1 (Monticelli and Rao, 2002). Yet, the respective human proteins belong to our LCC (**Figure 5**), thereby displaying about average to high connectivity.

Interestingly, the rodent orthologs of IL4 and of the second interleukin in our LCC, IL13, have been implicated in neuron survival, protection, and recovery (Pan et al., 2013; Walsh et al., 2015). IL4 and IL13 further share anti-inflammatory properties (Mori et al., 2016) and their common receptor comprises a subunit, IL13RA1 which has above-average connectivity in our network (**Figure 5**). The coding gene *IL13RA1* resides in the PD susceptibility locus PARK12 and its murine counterpart is expressed in dopaminergic neurons of the ventral tegmental area and the substantia nigra pars compacta (Morrison et al., 2012). Thus, also IL13RA1/Il13ra1 relegate to dopaminergic neurodegeneration.

Confirmation of a JAK/STAT-mediated implication of our network and especially of the LCC in neuroprotection and neurodegeneration comes from HSP90AA1 (also HSP90). Not only that this chaperone had the most direct PIPs in our LCC but HSP90AA1 also interacts with STAT3 in human cells (Sato et al., 2003) (**Figure 5**). This again stabilizes the folding of another protein in our network, i.e., the phosphatase PIM1 (Shen et al., 2014), which once more builds the bridge to neuron survival: Pim1 inhibition rescues Aβ and Tau pathology in murine brain (Velazquez et al., 2016), and inhibition of human PIM1 induces the neuroprotective transcription factor NFE2L2 (also NRF2; McMahon et al., 2014). Yet, NFE2L2 and its inhibitor KEAP1 (see Yamazaki et al., 2015) are further components of the present LCC (**Figure 5**).

### Post-transcriptional Expression Regulation

The present network and its LCC are additionally linked to gene silencing, namely through average- to highly connected proteins such as TARBP2, DICER1, and EIF2C1-4 (also AGO1-4) (**Figures 5**, **6**; see also Vernes et al., 2011). This pathway will affect the expression of a wide range of indirect FOXP2 targets but also gene silencing of *FOXP2* itself is in the range of possible. In support of the latter, *Foxp2* showed premature expression in the embryonic neocortex of mice whose *Dicer* gene was knocked out (Clovis et al., 2012; but see Haesler et al., 2007). Whether in the one direction or just the other way around balanced gene silencing is certainly important for normal development. This is demonstrated by deletions involving *EIF2C1* and *EIF2C3* which were recently reported to associate with facial dysmorphologies, speech and motor delay, and also with moderate intellectual disability (Tokita et al., 2015). In addition, DICER1 and EIF2C2 appear to be connected with the pathogenesis of Huntington’s disease (HD; e.g., Banez-Coronel et al., 2012; see also Batassa et al., 2010), thus providing an additional link between our LCC and dopamine imbalance (Chen et al., 2013).

The regulatory subunit PAN3 of the poly(A) nuclease PAN suggests an influence of our LCC on post-transcriptional expression regulation through mRNA decay (**Figures 5**, **6**) (Uchida et al., 2004). The LCC is additionally pertinent to ribosome recruitment (compare, e.g., Vernes et al., 2011), as exemplified by PAIP1, PAIP1-binding PABPC1, and the highly connected eukaryotic translation initiation factors EIF4E and EIF4G1 (e.g., Craig et al., 1998) (**Figure 5**). Yet, also ribosome recruitment is certainly vital for normal neural functioning as illustrated by late-onset motor incoordination in model mice upon sequestration of Pabpc1 (Damrath et al., 2012). Accordingly, mutations in *EIF4G1* have been recognized to associate with PD, and deregulation of *EIF4E* activity seems to increase susceptibility to autism (AUTS19) (Neves-Pereira et al., 2009; Chartier-Harlin et al., 2011).

Five moderately connected mitochondrial ribosomal subunits (MRPSs) underline that our LCC contributes to mitochondrial protein synthesis (**Figure 5**). The significance of this process for neuronal survival is exhibited by differential *MRPS6* levels in PD patients relative to unaffected individuals (Papapetropoulos et al., 2006). Moreover, a mutation in the *MRPS16* gene induces respiratory chain dysfunction with fatal consequences including agenesis of corpus callosum and death (Miller et al., 2004; Emdadul Haque et al., 2008). In further support of an effect of FOXP2 upon nervous system development through mitochondrial translation, murine isoform Foxp2Ex12+ has been localized to mitochondria in Purkinje cells – especially in cellular buds giving rise to dendrites (Tanabe et al., 2012). Yet, Purkinje cells have been reported to show altered synapse plasticity in mice carrying humanized *Foxp2* (Reimers-Kipping et al., 2011).

Mitochondrial translation also builds the bridge to another LCC member, namely ERP44 (also ERp44; **Figure 5**). This chaperone regulates, along with other proteins, the association of mitochondria with the endoplasmatic reticulum (ER). The establishment and maintenance of this interface is pivotal for cellular survival due to its influence on lipid transport, energy metabolism, and Ca^2+^ signaling (Hayashi et al., 2009). In particular, the latter implication might involve an inhibitory effect of ERP44 upon inositol-1,4,5-trisphosphate (IP3) receptors as demonstrated in mouse cerebellar microsomes (Higo et al., 2005). Yet, the implication of the LCC in phosphatidylinositol-mediated signaling was already mentioned, and Ca^2+^ release from the ER through IP3 receptors is altered in AD, HD, and ASD patients (see Schmunk et al., 2015 and references therein).

### Membrane Conductivity and Cell–Cell Adhesion

Present evidence for differential regulation of *GABRE* under hsa*FOXP2* control corroborates previous findings stressing the importance of GABAergic circuitry for the evolution of speech and language (e.g., Boeckx and Benítez-Burraco, 2014a,b) (**Figure 5** and **Table 2**). *GABRE* shows wide tissue distribution but appropriately spliced mRNA was exclusively detected in the hypothalamic region and hippocampus and, to a much lesser degree, in heart tissue (Whiting et al., 1999). Consequently, *GABRE* might have more importance for nervous system functioning than known to date. Besides GABRE, it is ZDHHC3 (also GODZ) which links our LCC with GABAergic wiring (**Figure 5**). The Golgi-specific DHHC zinc finger protein palmitoylates the γ2 subunit of GABA(A) receptors in neurons as demonstrated for the murine brain (Keller et al., 2004). Membrane conductivity is also modulated by present LCC member CFTR (**Figure 5**), which regulates chloride (and HCO3-) currents (Weyler et al., 1999). Interestingly, CFTR interacts with the abovementioned pERM and with another LCC member, i.e., SLC9A3R1 (also NHERF1) (**Figure 5**) (Alshafie et al., 2014). However, binding of SLC9A3R1 stabilizes the ERM complex and its kinase PTEN (Georgescu et al., 2014), whose neuronal and neural implications have been discussed above.

Importance for nervous system development through cell–cell adhesion has been found for diverse cadherins including CDH4 (also R-cadherin) which is encoded by one of the new FOXP2 targets (**Figures 3A**, **5** and **Table 2**; e.g., Oblander and Brady-Kalnay, 2010). Supporting the neural relevance of these Ca^2+^-dependent proteins, *CDH4* along with the gene coding for LCC member CDH11 (also OB-cadherin; **Figure 5**) was found to display differential spatial and temporal expression in developing marmoset brain (Matsunaga et al., 2015). In accordance, murine *Cdh4* and *Cdh11* seem to be essential for the association and migration of neurons during embryogenesis (Kimura et al., 1995; Hertel and Redies, 2011). Such relevance might partly reflect their interaction with other members of the cadherin family (e.g., Paulson et al., 2014). For instance, murine Cdh4 interacts with Cdh2 (N-cadherin) whose neuronal relevance is well-established (Matsunami et al., 1993). Moreover, *Cdh11* and *Cdh2* at least have overlapping functions including the regulation of β-catenin abundance and β-catenin-dependent gene expression (Di Benedetto et al., 2010). Yet, *Cdh2* is another Foxp2 target, whose regulation has been shown to affect the detachment of differentiating neurons from the neuroepithelium (e.g., Rousso et al., 2012).

FOXP2’s likely influence on neuronal development is further reflected by transmembrane LRRTM2, i.e., another member of the present LCC (**Figure 5**), which presumably regulates synapse formation through neurexin binding (Ko et al., 2009). An involvement in neurite formation and synapse formation is likewise probable for the transmembrane semaphorin SEMA6D (**Figure 5**) as suggested by observations in different model systems (e.g., Leslie et al., 2011). Congruously, the murine gene was previously reported to show Foxp2-driven expression regulation during neurite outgrowth (Vernes et al., 2011). It is thus unsurprising that SEMA6D matched with all neuron-related GO terms that were enriched in the present analysis, despite its peripheral position in our LCC (**Figure 5** and Supplementary Table 2.8).

## Conclusion

In the present study, we compared expression levels between SH-SY5Y cell lines stably overexpressing human *FOXP2* cDNA with cell lines stably transfected with *FOXP2* cDNAs of marmoset, macaque, and chimpanzee (**Figure 1**). Using RNA-seq, RT-qPCR, and Western blotting, we identified 13 new FOXP2 targets with differential expression levels under hsa*FOXP2* control (**Tables 2**, **3**; also **Figure 3**). The putative promoter sequences of all new target genes contained previously published FOXP2/FOXP2-binding motifs. Multiple matches of publicly available FOXP2-ChIP-seq reads with fragments inside the same promoter sequences additionally pointed to a potential direct binding of FOXP2. Thus, down-regulation of expression might reflect that hsaFOXP2 represses the respective target genes more efficiently than any of the non-human FOXP2s studied. The opposite might be true for transcription of *SEBOX*, the only gene amongst the new targets that showed hsaFOXP2-driven up-regulation. Whether their transcription is directly or indirectly regulated by FOXP2, the detection of 13 new targets denotes that the extent of the FOXP2-driven network is greater than currently known. It is further conceivable that the extent of the FOXP2-driven network was underestimated so far especially at the expense of target genes with moderate or even low transcription rates.

The 13 new FOXP2 targets, along with 27 reproduced ones set the start point for the reconstruction of a PPI network (**Figure 5**). The resulting network contained in total 80 proteins, thereof 43 with confirmed experimental evidence for FOXP2/Foxp2-driven expression regulation. Altogether 49 proteins in the network showed FOXP2/Foxp2-driven and/or songbird song-related expression regulation (**Figure 3**, **Tables 2**, **3**, and Supplementary Tables 2.1, 2.4; see also Spiteri et al., 2007; Vernes et al., 2007, 2011; Hilliard et al., 2012). In-depth literature screening and GO analysis underlined a general pattern showing that FOXP2 is effective also indirectly through signaling cascades and other transcriptionally and post-transcriptionally active proteins (**Figure 6** and Supplementary Table 2.8; also, e.g., Marcus and Fisher, 2003; Konopka et al., 2009). Additional functional domains whose fine-tuning might have had a considerable effect on hominization are as follows: regulation of cellular signaling and communication, protein and nucleotide metabolism and catabolism, as well as cellular migration, differentiation and development inclusively neuronal differentiation and survival (**Figure 6**). In particular, the neural and neuronal relevance of FOXP2 was demonstrated before (see, e.g., Enard et al., 2009; Konopka et al., 2009). However, the present study illustrates that also less connected proteins with only moderate to low expression levels can significantly alter our understanding of FOXP2’s role in neural and neuronal development, maintenance, and functioning: Thus, GO terms including the words “neuron” or “axonogenesis” (thus excluding “neurotrophin”) only appeared to be enriched as long as the 13 new FOXP2 targets were included (compare Supplementary Tables 2.8, 3.3). Nonetheless, the numerous connections between the present network and neuritogenesis, neuron differentiation, etc. were by no means restricted to the new FOXP2 targets (see Discussion for details).

It is further worthwhile that we identified comparably few genes (see present Supplementary Table 2.1: genes without significant support) that were previously reported as differentially expressed between SH-SY5Y cells overexpressing either human *FOXP2* cDNA or a “chimpanized” variant (*FOXP2*^chimp^; see Konopka et al., 2009, their Supplementary Table 1). The same applies with respect to an earlier examination of the effect of the two human-specific aa substitutions in mice carrying humanized *Foxp2* (*Foxp2*^hum^; see Enard et al., 2009; their Figures S8A,B, right panel). On the contrary, the overlap was much higher between the present protein sample and the lists of targets that were identified by FOXP2-ChIP-seq in human tissues and in SH-SY5Y cells stably overexpressing human FOXP2 (Spiteri et al., 2007, their Table 1; Vernes et al., 2007; their Table 1). The overlap increased when expanding the comparison to Foxp2 targets identified by ChIP-seq in wild type murine brain (Vernes et al., 2011, their Table S1). The number of reproduced loci further rose when genes with songbird song-related expression regulation were considered (Hilliard et al., 2012, their Table S2). These differences in overlap might partially reflect the different size of the gene lists taken as references. Nonetheless, there seems to be a trend displaying that we primarily re-identified genes from the studies that used non-mutated cDNAs rather than mutated ones combining states of two species. From our point of view this supports the suitability of species-specific cDNAs with counterparts in nature for studying FOXP2’s role in evolution.

The present study differs from others especially with respect to the phylogenetic concept applied. Yet, this conceptual extension is not only of theoretical value as illustrated by the special case of *PHOX2B*. This gene was already a candidate for FOXP2-mediated expression regulation in a previous study which compared expression levels in SH-SY5Y cells overexpressing human FOXP2 *versus* cells carrying empty vector (Spiteri et al., 2007). Although RT-qPCR indicated down-regulation of transcription in the overexpressing cells the difference was not significant. In contrast, the present approach yielded significant support for down-regulation of *PHOX2B* expression in hsa*FOXP2*-overexpressing cells relative to cells overexpressing ptr*FOXP2* and mmu*FOXP2*. In our opinion this illustrates the usefulness of a phylogenetic approach including at least one additional non-human model besides human and chimpanzee models in order to unmask changes in the fine-tuning of target gene expression that might have importance for human evolution and health.

In this way, we determined multiple connections of the FOXP2-driven network and its LCC to developmental (ASD, SCZD, Down syndrome, agenesis of corpus callosum, trismus-pseudocamptodactyly, ankyloglossia, facial dysmorphology) and neurodegenerative disorders and diseases (AD, PD, HD, LBD, ALS), deafness, and dyslexia (for details, see Discussion). In particular, the links to AD, PD, and HD pathologies but also diverse connections to the affected neuron types and brain regions substantiate the importance of FOXP2 for dopaminergic wiring and neurodegeneration (see Discussion for details; also, e.g., Reimers-Kipping et al., 2011; Hilliard et al., 2012; Devanna et al., 2014; Pfenning et al., 2014; Schreiweis et al., 2014). Moreover, reported communication deficits in at least some cases of AD, PD, HD, LBD, ALS, ASD, SCZD, and Down syndrome (Murray, 2000; Yoder and Warren, 2004; Stephane et al., 2007; Abrahams and Geschwind, 2010; Kupferberg, 2010; Reilly et al., 2010; Ferris and Farlow, 2013) confirm the well-established involvement of FOXP2 in the evolutionary and developmental acquisition of speech and language (see, e.g., Vernes and Fisher, 2009; Bolhuis et al., 2010; Enard, 2011; but see Mallick et al., 2016).

However, the present approach did not only confirm and substantiate previous knowledge. Thus, we were able to delineate new pathways of how human FOXP2 governs neurogenesis, neurite outgrowth, synapse plasticity, neuron migration, and the regulation of conductivity. These involve: (i) transcription regulation through NURR1, PHOX2B, TBX22, SEBOX, and FOXL1, (ii) cadherin-mediated cell–cell adhesion (CDH4, CDH11), (iii) gene silencing through DICER1 and RISC, (iv) JAK/STAT signaling and neuro-inflammation, and (v) the organization of the microtubule (DCDC2, KIF13B), myosin (MYH8, MYH13), and actin cytoskeleton (PTPRQ, MSN and ERM complex). Single interactors of gene silencing, the ERM complex and JAK/STAT signaling also appeared in other FOXP2-directed studies (e.g., RDX in Figure 3 of Konopka et al., 2009; Dicer1 and Jak1 in Table S1 of Vernes et al., 2011). Yet, such implications of FOXP2 seemingly did not emerge with the same clarity before. In this way, we regard also gene silencing, JAK/STAT signaling, and the regulation of the ERM complex as novel FOXP2-driven pathways.

We hope that these novel insights may open up new avenues toward a better understanding of the molecular causes of the aforementioned developmental disorders, of communication deficits and especially of neurodegenerative diseases. With respect to the latter it would be advantageous to further investigate if down-regulation of newly detected FOXP2 targets such as *DCDC2*, *MYH8*, and *MYH13* under hsa*FOXP2* control is due to direct FOXP2-binding. FOXP2-ChIP-qPCR could be a good way to answer this question, and also for validating the expressional differences which we observed in RNA-seq, RT-qPCR, and Western blot analyses. The entire spectrum of techniques could further be applied to transiently transfected SH-SY5Y cells, which overexpress different primate FOXP2 cDNAs. Reproduction of our findings in such cell lines would rule out that inestimable effects of the foreign DNA integrates (pcDNA3-constructs) into the genomes of SH-SY5Y cells have biased our results. This seems especially relevant considering that the integration sites and the number of integrated plasmids can vary between stably transfected cells and their descendants, due to the random integration of plasmids (e.g., Mitin et al., 2001). In genes such as *PHOX2B* and *NURR1* further steps could involve animal studies to verify if their established implication in brain development and maintenance is FOXP2-driven or not. Lastly, in cases where the present study evidenced down-regulated expression at the protein level (CDH4, MSN, BACE2) the next steps could involve the investigation of murine knock-outs against the background of neurodegenerative disease phenotypes. Preliminary data on *Cdh4* seem promising in this respect: A viable knock-out reportedly decreased activity, amongst others (see MGI:99218). However, if this change ultimately reflects changes in *Foxp2* expression or Foxp2 activity and if the behavioral data associate with an alteration in neuronal wiring are questions waiting for an answer.

## Author Contributions

FO, PK, and AR generated the pcDNA3-*FOXP2* constructs and cultivated, transfected, and characterized HEK293 and SH-SY5Y cells. The same authors carried out immunoblotting and densitometric analysis. BH, UZ, and HH conducted and analyzed RT-qPCR measurements. DR and HH analyzed RNA-seq data as well as previously published FOXP2-ChIP-seq data, and searched putative target gene promoters for established FOXP2/FOXP2-binding motifs. FB assisted in data analysis. HH conducted network reconstruction, gene ontology analysis, in-depth literature screening, and evolutionary analysis. HH, UZ, FO, and SR conceived the study. HH wrote the manuscript. SR, FO, UZ, DR, and FB co-wrote the manuscript. All authors were involved in the scientific interpretation of the results.

## Conflict of Interest Statement

The authors declare that the research was conducted in the absence of any commercial or financial relationships that could be construed as a potential conflict of interest.

## Footnotes

1 https://github.com/alexdobin/STAR/releases

2 https://genome.ucsc.edu/

3 www.hartfaalcentrum.nl

4 https://imagej.nih.gov/ij/index.html

5 http://string-db.org

6 www.cytoscape.org

7 brainspan.org
