# Supplementary material for: The FOXP2-Driven Network in Developmental Disorders and Neurodegeneration
Source: Front Cell Neurosci. 2017 Jul 26;11:212. doi: 10.3389/fncel.2017.00212 (PMC5526973; doi:10.3389/fncel.2017.00212)
Supplement: Supplementary file 4 [file Image_1.pdf]

SUPPLEMENTARY IMAGE 1

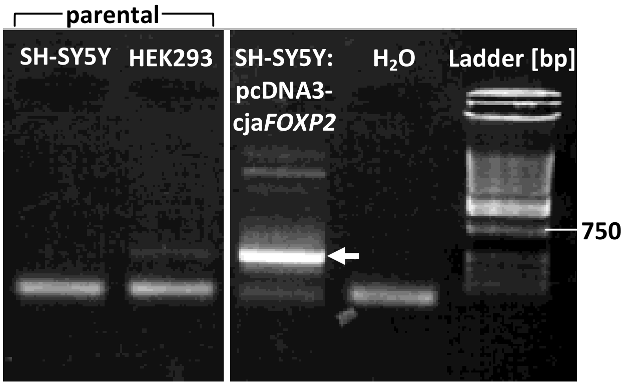

SUPPLEMENTARY IMAGE 1 | Results of *FOXP2*-RT-PCR after separation by agarose gel electrophoresis. Pure water was used as a negative control. SH-SY5Y cells that were stably transfected with pcDNA3-cja*FOXP2* served as a positive control for exogenous *FOXP2* transcription (arrow). Transcription of endogenous *FOXP2* is absent in parental SH-SY5Y cells and very low in parental HEK293 cells. This indicates the suitability of both strains for i) analysis of the functionality of pcDNA3-*FOXP2* constructs in transiently transfected HEK293 cells and ii) studying target gene expression levels under control of exogenous *FOXP2* in stably transfected SH-SY5Y cells. Low-sized bands represent unbound primers (see Material and Methods for primers used). DNA was stained with ethidium bromide.
