# Supplementary material for: The FOXP2-Driven Network in Developmental Disorders and Neurodegeneration
Source: Front Cell Neurosci. 2017 Jul 26;11:212. doi: 10.3389/fncel.2017.00212 (PMC5526973; doi:10.3389/fncel.2017.00212)
Supplement: Supplementary file 5 [file Image_2.pdf]

SUPPLEMENTARY IMAGE 2

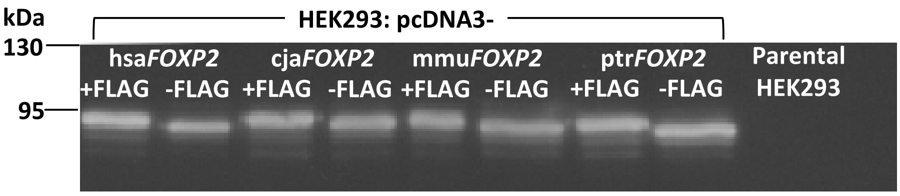

SUPPLEMENTARY IMAGE 2 | Western blot of SDS-PAGE-separated lysates from parental and transiently transfected HEK293 cells. Polyclonal anti-FOXP2 detected no endogenous FOXP2 protein in the lysate prepared from parental cells. However, distinct bands indicate exogenous expression in HEK293 cells transiently transfected with pcDNA3 constructs, which contained alternative primate *FOXP2* cDNAs. Higher molecular weight of protein in cell lines overexpressing exogenous FOXP2 with FLAG tag (+FLAG) than in cell lines producing FOXP2 without FLAG tag (-FLAG) demonstrates transcription of full length exogenous cDNAs. cja, marmoset (*Callithrix jacchus*); hsa, human (*Homo sapiens*); mmu, Rhesus monkey (*Macaca mulatta*); ptr, chimpanzee (*Pan troglodytes*).
