# Supplementary material for: The FOXP2-Driven Network in Developmental Disorders and Neurodegeneration
Source: Front Cell Neurosci. 2017 Jul 26;11:212. doi: 10.3389/fncel.2017.00212 (PMC5526973; doi:10.3389/fncel.2017.00212)
Supplement: Supplementary file 6 [file Image_3.pdf]

SUPPLEMENTARY IMAGE 3

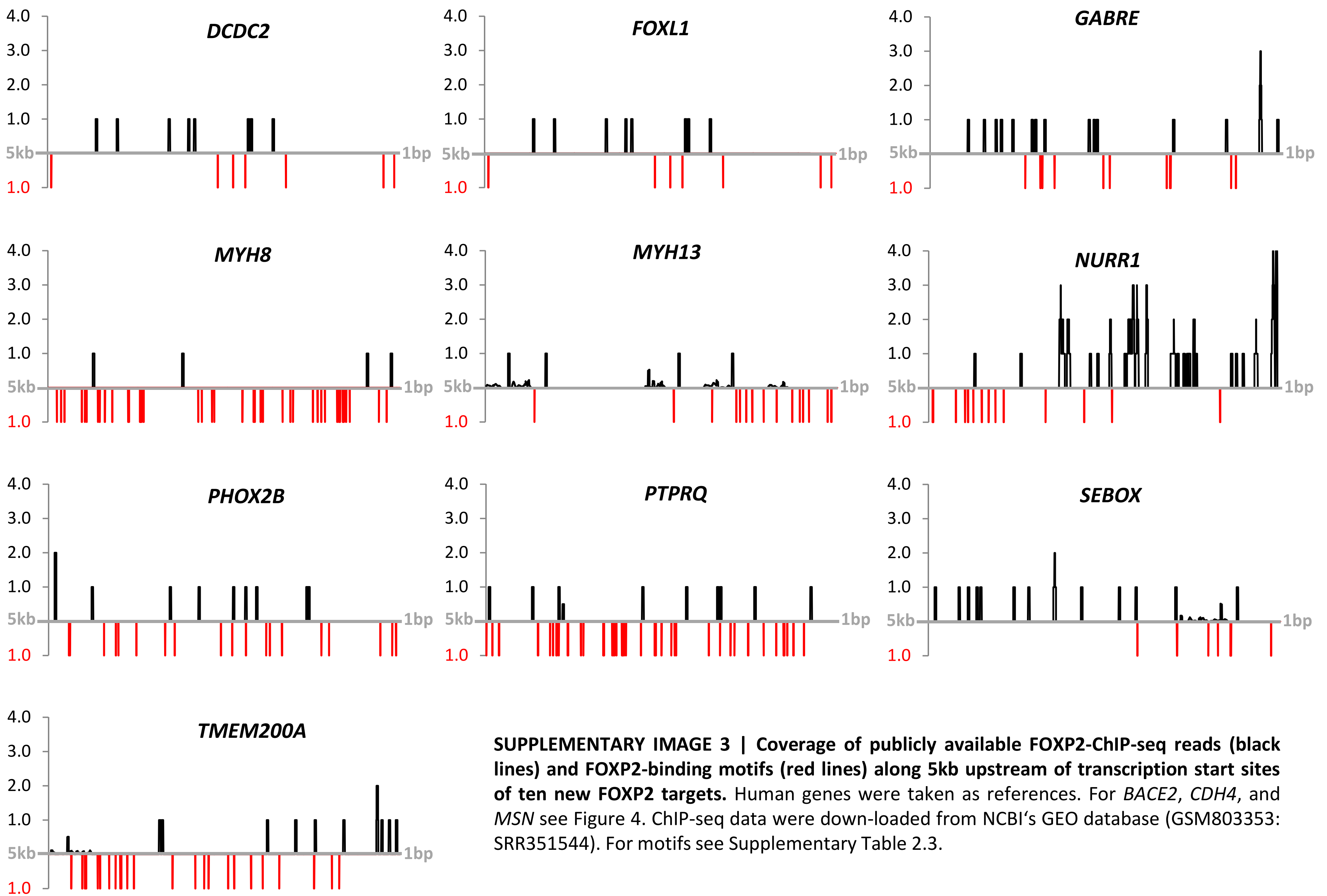

SUPPLEMENTARY IMAGE 3 | Coverage of publicly available FOXP2-ChIP-seq reads (black lines) and FOXP2-binding motifs (red lines) along 5kb upstream of transcription start sites of ten new FOXP2 targets. Human genes were taken as references. For *BACE2*, *CDH4*, and *MSN* see Figure 4. ChIP-seq data were down-loaded from NCBI's GEO database (GSM803353: SRR351544). For motifs see Supplementary Table 2.3.
